# Unified iontronic sensing for operando monitoring of physical-chemical events in lithium-ion batteries

**DOI:** 10.1093/nsr/nwaf151

**Published:** 2025-04-25

**Authors:** Yu Chang, Yu Cheng, Rui Jia, Ruojiang Wang, Qing Xu, Lanqing Gong, Yike Wei, Bin Tang, Chenhui Guo, Bin Sun, Xingmin He, Xueyan Li, Lili Gong, Hong Ye, Xiaoyang Wang, Yitao Dai, Mingdong Dong, Yongbing Tang, Fan Zhang, Peng Tan, Tingrui Pan

**Affiliations:** Department of Precision Machinery and Precision Instrumentation, University of Science and Technology of China, Hefei 230026, China; Center for Intelligent Medical Equipment and Devices (iMED), Suzhou Institute for Advanced Research, University of Science and Technology of China, Suzhou 215123, China; Bionic Sensing and Intelligence Center (BSIC), Institute of Biomedical and Health Engineering, Shenzhen Institute of Advanced Technology, Chinese Academy of Sciences, Shenzhen 518055, China; Advanced Energy Storage Technology Research Center, Shenzhen Institute of Advanced Technology, Chinese Academy of Sciences, Shenzhen 518055, China; Dongguan Key Laboratory of Interdisciplinary Science for Advanced Materials and Large-Scale Scientific Facilities, School of Physical Sciences, Great Bay University, Dongguan 523000, China; Department of Precision Machinery and Precision Instrumentation, University of Science and Technology of China, Hefei 230026, China; Center for Intelligent Medical Equipment and Devices (iMED), Suzhou Institute for Advanced Research, University of Science and Technology of China, Suzhou 215123, China; Department of Precision Machinery and Precision Instrumentation, University of Science and Technology of China, Hefei 230026, China; Center for Intelligent Medical Equipment and Devices (iMED), Suzhou Institute for Advanced Research, University of Science and Technology of China, Suzhou 215123, China; Department of Precision Machinery and Precision Instrumentation, University of Science and Technology of China, Hefei 230026, China; Center for Intelligent Medical Equipment and Devices (iMED), Suzhou Institute for Advanced Research, University of Science and Technology of China, Suzhou 215123, China; Advanced Energy Storage Technology Research Center, Shenzhen Institute of Advanced Technology, Chinese Academy of Sciences, Shenzhen 518055, China; Advanced Energy Storage Technology Research Center, Shenzhen Institute of Advanced Technology, Chinese Academy of Sciences, Shenzhen 518055, China; Bionic Sensing and Intelligence Center (BSIC), Institute of Biomedical and Health Engineering, Shenzhen Institute of Advanced Technology, Chinese Academy of Sciences, Shenzhen 518055, China; Bionic Sensing and Intelligence Center (BSIC), Institute of Biomedical and Health Engineering, Shenzhen Institute of Advanced Technology, Chinese Academy of Sciences, Shenzhen 518055, China; Department of Thermal Science and Energy Engineering, University of Science and Technology of China, Hefei 230026, China; Department of Thermal Science and Energy Engineering, University of Science and Technology of China, Hefei 230026, China; Department of Thermal Science and Energy Engineering, University of Science and Technology of China, Hefei 230026, China; TacSense Technology (Shenzhen) Co. Ltd., Shenzhen 518055, China; TacSense Technology (Shenzhen) Co. Ltd., Shenzhen 518055, China; Center for Intelligent Medical Equipment and Devices (iMED), Suzhou Institute for Advanced Research, University of Science and Technology of China, Suzhou 215123, China; Interdisciplinary Nanoscience Center (iNANO), Aarhus University, Aarhus C DK-8000, Denmark; Advanced Energy Storage Technology Research Center, Shenzhen Institute of Advanced Technology, Chinese Academy of Sciences, Shenzhen 518055, China; Advanced Energy Storage Technology Research Center, Shenzhen Institute of Advanced Technology, Chinese Academy of Sciences, Shenzhen 518055, China; Department of Thermal Science and Energy Engineering, University of Science and Technology of China, Hefei 230026, China; Department of Precision Machinery and Precision Instrumentation, University of Science and Technology of China, Hefei 230026, China; Center for Intelligent Medical Equipment and Devices (iMED), Suzhou Institute for Advanced Research, University of Science and Technology of China, Suzhou 215123, China

**Keywords:** iontronic, flexible electronics, lithium-ion battery, operando sensing, implanted sensor, battery safety

## Abstract

Operando measurement of expansion force has become an effective way of monitoring physical-chemical events in lithium-ion batteries, of which the conventional optical means is limited by material fragility, structural incompatibility and system complexity. The utilization of flexible sensors can potentially address these challenges; however, their functionality and stability are restricted within hours in corrosive environments. Here, an *in-situ* unified iontronic sensing mechanism, derived from the super-capacitive electrode/electrolyte interface, is developed for expansion-force measurement in highly corrosive electrolyte environments. Specifically, it leverages the structural and material similarities between batteries and iontronic sensors to create a unified assessing device, utilizing only existing materials to produce *in-situ* detection. Consequently, the intelligence-incorporated architecture exhibits exceptional stability for continuous measurements over one month, enabling 400 charging/discharging cycles in a battery lifespan. Therefore, the unified iontronic sensing device, which has long-term on-board monitoring of expansion forces, offers an accurate and effective solution for the aging evaluation and safety pre-warning of lithium-ion batteries.

## Teaser

An in-situ pressure sensing mechanism, which leverages the structural and material similarities between batteries and iontronic sensors, is developed to create a unified assessing device utilizing only the existing materials for achieving long-term operando monitoring of physical-chemical events in lithium-ion batteries.

## INTRODUCTION

Lithium-ion batteries (LIBs) are widely considered the optimal choice for electric vehicles (EVs) and energy storage applications due to their inherent advantages in terms of superior energy density, substantial power density, robust cycling stability and extended service life [1]. However, the extended charging time, which is limited by the constrained electrochemical reaction rate at the electrodes, poses a significant obstacle to the broader application of LIBs, particularly in the field of EVs [2]. Therefore, enhancing the fast-charging capability of LIBs is highly desirable. However, fast-charging technology always faces the challenge of lithium (Li) plating on the anode, which significantly accelerates capacity degradation and impacts battery safety [3]. For instance, a high charging rate (C-rate) can potentially lead to Li plating instead of the intercalation of lithium ions (Li^+^ ions) in the anode materials. The exposed Li metal will generate an excess of solid electrolyte interphase (SEI) upon contact with the electrolyte. Additionally, certain portions of Li deposits become disconnected from the anode and transform into ‘dead Li’, which does not participate in the discharge reaction. Both of these factors consume additional active Li, resulting in significant capacity loss [4]. More importantly, Li tends to form dendrites, which might penetrate the separator and cause an internal short-circuit, resulting in thermal runaway or fire [5]. Therefore, monitoring these physical-chemical phenomena is crucial for their onboard application to achieve a balance between safety, aging and charging at high rates [6].

Conventional monitoring of voltage, current and temperature in a battery management system cannot effectively detect early stage Li plating [7]. Recently, the measurement of pressure changes during charging/discharging has been validated as an effective approach for monitoring Li plating [8]. The Li plating phenomena, in theory, can result in abnormal expansion of the LIB electrodes, thereby causing pressure fluctuations within the battery. Typically, the measurement is performed externally by directly attaching a pressure gauge [9,10]. However, the rigid nature of the metal package, commonly in square or cylindrical shapes, significantly impacts the pressure detection in the package, as well as the sensitivity and accuracy of the sensor. Consequently, embedded pressure sensors have gained considerable attention for real-time monitoring of the reactions [11]. Fiber Bragg grating (FBG) sensors, which possess long-term stability in corrosive battery electrolytes, have been integrated into batteries to monitor stress variations [12,13]. Even so, the practical implementation of FBG sensors poses several challenges. First, a complicated optical demodulation system is required, resulting in increased dimensions, weight and cost. Besides, the compatibility issues between FBG implantation and battery fabrication processes can disrupt the structural integrity of the battery. Moreover, due to the fragile nature of the FBG material, there is a risk of fracture failure. The development of flexible electronics has introduced an alternative approach due to their distinct advantages. For instance, electric signal readout systems are simple and cost-effective. These sensors possess the same membranous format as the electrodes of LIBs. Moreover, the robust nature of polymer-based materials used in flexible sensors ensures their resistance to fracture or deformation in challenging application environments [14,15]. Flexible strain sensors have been embedded into LIBs for detection of the strain caused by electrode expansion. But such strain is significantly influenced by the shape and stiffness of the package. Additionally, flexible pressure sensors can directly detect pressure variations in the LIB. However, a significant challenge arises from the corrosive nature of the battery's electrolyte environment, which has a substantial impact on the properties of the sensing materials upon contact. Despite efforts to employ corrosion-resistant encapsulation, completely preventing electrolyte penetration remains challenging due to both the non-zero transmission rate of the electrolyte in the encapsulation and the exceptionally long service life of the battery [16,17].

The rise of iontronic sensing technology provides a potential solution to address this issue. The iontronic pressure sensor is designed with a structurally deformable super-capacitive interface in response to external mechanical stimuli. The application of external pressure enhances the contact area of the interface, resulting in an increase in the capacitance of the sensor [18–20]. It is worth noting that iontronic sensing devices share similar device structures and building materials with LIBs. Given the ubiquitous presence and accessibility of ionic carriers in nature, only conductive surfaces are fundamentally required to establish the iontronic interface for pressure sensing [21–23]. For example, human skin, plants and even aquatic environments have been proposed as natural ionics that participate in the formation of iontronic sensors, i.e. environment-unified iontronic sensors. Specifically, this proposed simple sensing architecture does not necessitate encapsulation and allows seamless integration with the surrounding environment.

**Figure 1.**
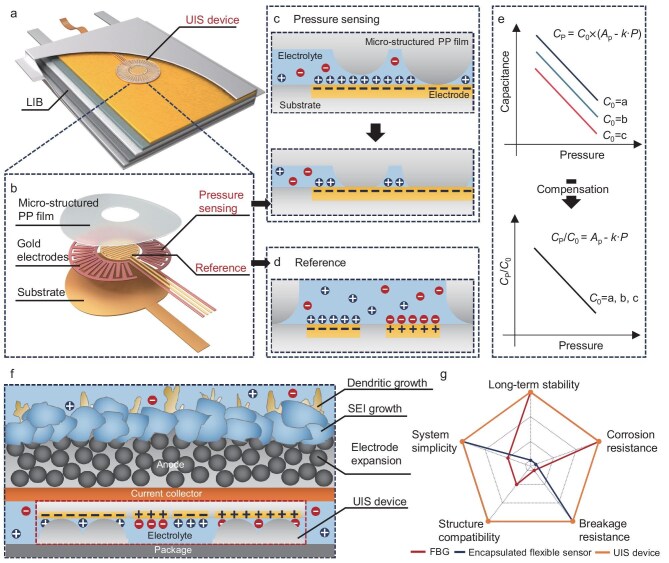
Structure and operation principle of the *in-situ* unified iontronic sensing (UIS) device. (a) Scheme of the pouch cell with *in-situ* UIS device for pressure monitoring. (b) Structure and building materials of the UIS device. (c) Operation principle for pressure sensing of the UIS device. (d) Operation principle of the reference unit, which measures the unit area capacitance (UAC) of the electrode/electrolyte interface. (e) Theoretical explanation of the compensation for pressure sensing using the reference unit. By employing a normalized parameter, the influences caused by varying UACs of the electrolytes can be effectively addressed in theory. (f) Scheme of the UIS device in the lithium-ion battery (LIB) for pressure variation detection, which is related to the physical-chemical events of electrode expansion, SEI growth and Li dendritic growth. (g) Performance comparisons of the FBG sensor, encapsulated flexible sensor and UIS device for operando pressure sensing in LIBs.

For the purpose of operando monitoring of physical-chemical events associated with pressure variation in LIBs with high stability and accuracy, an emerging flexible sensing mechanism called *in-situ* unified iontronic sensing (UIS) has been developed for pressure measurement in highly corrosive liquid environments. Specifically, the UIS mechanism leverages the structural and material similarities between LIBs and iontronic sensing devices to create an *in-situ* UIS device that utilizes existing organic electrolytes and building materials within LIBs to form the sensing structure. Based on the UIS device, an intelligent unified battery is obtained that integrates high power supply with *in-situ* pressure sensing capabilities (Fig. 1a). The UIS device comprises two independent units integrated into a compact layered architecture, as depicted in Fig. 1b. The pressure sensing unit detects pressure variations through capacitive changes at the pressure-sensitive electrolyte/electrode interface (Fig. 1c). Additionally, a reference electrode directly exposed to the electrolyte is incorporated into the UIS device to enable real-time monitoring of ionic conductivity (Fig. 1d) and compensate for the influence of varying electrical properties of the electrolyte on pressure sensing, as illustrated in Fig. 1e. When embedded into a LIB, the UIS device is positioned between the package and the outermost current collector at the anode for the operando detection of pressure variations, which enables monitoring of various physical-chemical phenomena during operation, including electrode expansion, SEI growth and dendritic growth, as illustrated in Fig. 1f. Figure 1g presents a comprehensive comparison of the performance of the FBG sensor, the encapsulated flexible sensor and the UIS device, with regard to operando pressure monitoring in LIBs. The UIS device, constructed from ductile materials and featuring a membranous structure, demonstrates remarkable system simplicity, structural compatibility with LIBs, and exceptional resistance to breakage on building materials. Moreover, owing to the utilization of battery electrolyte as the functional material, and to its components exhibiting long-term stability within LIBs due to shared building materials, this device has showcased outstanding long-term stability exceeding 1 month, as well as corrosion resistance in the LIB, enabling more than 400 charging/discharging cycles that cover the entire lifespan of the battery.

## RESULTS AND DISCUSSION

### Structural design and operation principles of the UIS device

For the purpose of operando pressure measurements in LIBs, the UIS device is designed with two distinct modules. Specifically, as depicted in Fig. 1b and Fig. S1, the device comprises a reference unit that records the ionic conductivity of the electrolyte in the central region, and a pressure sensing unit positioned adjacent to the reference unit. Both units are integrated into a compact membranous structure consisting of three layers: a polyimide (PI) substrate layer, a gold electrode layer for each unit, and a micro-structured polypropylene (PP) film with a central aperture to expose the reference unit. Importantly, most materials utilized in the UIS device originate from existing components within LIBs. Specifically, PI serves as a tape substrate for securing multilayered current collectors/separators in the LIB [24], PP is widely employed as a separator material [25], and gold is one of the most chemically inert metals [26].

The pressure sensing unit consists of a ring-shaped interdigital electrode covered with a micro-structured PP film. Once embedded into the pouch cell, the electrolyte permeates into the gap and forms an iontronic interface with the interdigital electrode. As illustrated in Fig. 1c, when the external pressure on the pressure sensing unit increases, the micro-structures on the PP surface undergo elastic deformation, thereby squeezing out electrolyte from the interface. Correspondingly, the contact area between the interdigital electrode and the electrolyte decreases, leading to a decrease in the capacitive output of the unit. For an elastomer film with randomly distributed micro-structures on its surface, the capacitive output *C*_P_ of the pressure sensing unit under different external pressures P can be expressed as:


(1) \begin{eqnarray*}
{C}_{\mathrm{P}} = {C}_0 \times \ \left( {{A}_{\mathrm{P}} - k\frac{P}{E}} \right),
\end{eqnarray*}

where *C*_0_ is the unit area capacitance (UAC) of the iontronic interface between the interdigital electrode and the electrolyte, *A*_P_ represents the area of the interdigital electrode, *E* means the Young’s modulus of the PP film, and *k* refers to a constant associated with the surface morphology of the PP film. As can be seen, the capacitive output of the pressure sensing unit exhibits a negative linear correlation with the external pressure, while being positively influenced by the UAC of the iontronic interface.

According to Equation (1), the capacitive output of the pressure sensing unit is directly influenced by *C*_0_. Considering practical scenarios where an UIS device is embedded into different LIBs, variations in electrolyte composition and concentration, as well as temperature fluctuations, can all impact the ionic conductivity of the electrolyte, which is reflected by alterations in UAC. To compensate for the influence of varying UAC on pressure sensing accuracy, a reference unit (Fig. 1d) is introduced to detect the UAC of the electrolyte. The relationship between a normalized parameter *S*, derived from the output of the pressure sensing unit divided by that of the reference unit, and the external pressure *P*, can be expressed as:


(2) \begin{eqnarray*}
S &=& \frac{{{C}_{\mathrm{P}}}}{{{C}_{\mathrm{R}}}} = \frac{{{C}_0 \times \ ({A}_{\mathrm{P}} - kP)}}{{{C}_0 \times {A}_{\mathrm{R}}}} = \frac{{{A}_{\mathrm{P}} - kP}}{{{A}_{\mathrm{R}}}}\\
&=& a - bP.
\end{eqnarray*}

Therefore, the external pressure *P* can be determined from Equation (2), independent of the varying UAC of the electrolyte, as shown in Fig. 1e.

### Performance of the pressure sensing unit

**Figure 2.**
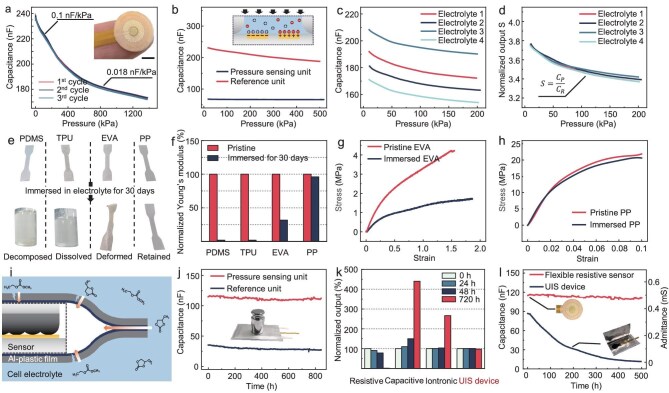
Sensing performance and stability of the UIS device. (a) Capacitance-to-pressure curve of the pressure sensing unit. The inset image shows the photo of the UIS device, and the scale bar is 3 mm. (b) The insensitivity to pressure of the reference unit. (c) Capacitance-to-pressure response curves detected in different electrolytes with varied ionic conductivities (adjusted by concentration and temperature, electrolyte 1: 2%, 30°C; electrolyte 2: 1%, 30°C; electrolyte 3: 3%, 30°C; electrolyte 4: 1%, 25°C). (d) Compensation of the varied ionic conductivity of the electrolyte for pressure sensing. (e, f) Stability of common elastomers for the flexible pressure sensor immersed in the battery electrolyte for 30 days. (g) Strain-stress curves of the EVA elastomer before and after immersion. (h) Strain-stress curves of the PP films before and after immersion. (i) Scheme of the penetration of the solvent molecule in the electrolyte through the defects in the metal package film and the polymer adhesive at the edges, influencing the performance of the encapsulated flexible pressure sensor. (j) Long-term stability of the UIS device immersed in the battery electrolyte. (k, l) Comparison of long-term stability between the pressure sensing unit of the UIS device and the encapsulated flexible pressure sensors based on resistive, capacitive and iontronic mechanisms in the battery electrolyte environment.

The mechanical performance of the pressure sensing unit, including the sensitivity, linearity and sensing range, can be evaluated based on the capacitance-to-pressure curve, as depicted in Fig. 2a. The pressure sensing unit demonstrates a high sensitivity of 0.1 nF/kPa in a range within 200 kPa, which is at least three orders of magnitude greater than that of conventional capacitive sensors [27], ensuring the accurate detection of minute pressure variations in environments with high electromagnetic noise, akin to those encountered during the charging/discharging processes of LIBs. Additionally, the curve exhibits a high linearity exceeding 0.99, fitting well with the theoretical analysis and facilitating device calibration while simplifying the readout circuit design. As the external pressure increases, the sensitivity of the device is reduced due to a decrease in the deformation ability of the micro-structures on the PP film. Specifically, the pressure sensing unit exhibits a sensitivity of 0.018 nF/kPa within a range of 400–1300 kPa, which is still detectable compared with the background capacitive noise of ∼1 pF. Furthermore, three consecutive loading experiments demonstrated that the mechanical response of the device has high repeatability, with variations lower than 0.5%. This indicates both excellent resistance to plastic deformation for the PP material and stability at iontronic interfaces under pressures as high as 1300 kPa, which are essential for achieving highly accurate pressure detection. More characterizations on the pressure sensing unit are shown in Fig. S2.

### Performance of the reference unit and compensation for pressure sensing

The electric conductivity of the electrolyte varies due to changes in its formulation, environmental temperature and aging of the LIB. These variations can affect the accuracy of pressure measurements taken by the sensing unit. Therefore, a reference unit is introduced to compensate for the changing electrical properties of the electrolyte on the pressure sensing unit by recording the real-time UAC of the electrolyte (Fig. S3). A test is conducted to verify the impact of pressure changes on the reference unit. As illustrated in Fig. 2b, while the capacitance of the pressure sensing unit decreases with increasing pressure, no discernible response is observed from the reference unit, ensuring accurate UAC detection under varying pressures. Figure 2c shows the capacitance-to-pressure curves of the pressure sensing unit in four different electrolytes with varying UACs. It is evident that there are distinct variations in the mechanical response across different electrolytes, indicating a varying correspondence between the external pressure and sensor output due to the varied UAC of the electrolyte. However, employing a compensation operation according to Equation (2) effectively mitigates the drifts among various UAC-dependent response curves, resulting in a consolidated response curve with only a marginal variation less than 3%, as depicted in Fig. 2d. This ensures the applicability of the UIS device for LIBs using different electrolytes or for LIBs with various UACs of electrolyte during charging/discharging or aging. Moreover, we have investigated the performance of the UIS device, including its mechanical response curves and self-compensation ability, within a temperature range of 5°C to 60°C, which covers the optimal operating temperature range for LIBs. The results are presented in Fig. S4. Although the capacitance-to-pressure curves of the pressure unit vary at different temperatures, the compensation operation can still minimize the influence of temperature on the conductivity of the electrolyte, achieving stable and accurate pressure detection even in a wide temperature range.

### Long-term stability of the UIS device in corrosive organic electrolyte

The performance of the UIS device relies heavily on the mechanical stability of the micro-structured PP film, as indicated by Equation (1). Generally, numerous elastomers are available for use in flexible pressure sensors as the pressure-sensitive layer. However, when utilized in organic electrolytes such as those commonly found in LIBs, comprising LiPF_6_ salt and carbonic ester solvent, most elastomers are prone to failure. In this study, four representative elastomers, polydimethylsiloxane (PDMS, silicones), thermoplastic urethane (TPU, polyurethanes), ethylene vinyl acetate copolymer (EVA, amorphous polyolefins) and PP (crystalline polyolefins), are immersed in an organic electrolyte for 30 days to evaluate their stability. Figure 2e and f and Fig. S5 illustrate the results, indicating that the PDMS sample decomposes due to corrosion caused by hydrofluoric acid resulting from the reaction between LiPF_6_ and trace water in the electrolyte [28]. Similarly, the TPU sample dissolves in carbonate ester solvent owing to its comparable polarities [29]. Furthermore, the EVA sample undergoes significant deformation due to swelling of its amorphous molecular chains [30], leading to a decrease in the modulus from 2.42 MPa to 0.77 MPa, as shown in Fig. 2g. In contrast, only the PP film retains its original state without any deformation or obvious changes on the Young`s modulus because of its compact crystal structure, which resists penetration of the polar solvent [31], as depicted in Fig. 2h. Notably, the use of non-ionic elastomers in the UIS device is not limited to PP, but it is the optimal choice due to its proven long-term stability as a constituent material in commercial LIB separators.

Considering the extended service life of LIBs, ensuring the long-term stability of embedded sensors becomes crucial. For current flexible pressure sensors, which employ piezoresistive, piezocapacitive, piezoelectric or iontronic mechanisms, their long-term stability relies heavily on the electrical and mechanical properties of functional materials. However, when in contact with LIB electrolyte, ions, corrosive components or organic solvents in the electrolyte may profoundly impact these properties. For instance, the commonly used carbonate ester solvent in LIB electrolytes tends to adsorb on carbon materials due to the attractive forces between polar groups, leading to variations in the resistivity of carbon materials, which are extensively employed as functional materials in piezoresistive sensors [32,33]. The encapsulation of the sensor effectively prevents direct contact with the electrolyte and extends its stable period within the electrolyte. Typically, metal foil provides the highest barrier efficiency for the electrolyte as a flexible packaging material, which is why current pouch cells use Al-plastic film as an encapsulation to prevent oxygen and moisture [34,35] (Table S1). However, small molecules in organic electrolytes, such as carbonate ester molecules, can still transmit through defects in the metal film and the polymer adhesive at the edges of the encapsulation [36,37], ultimately affecting sensor performance, as shown in Fig. 2i. In contrast, the UIS device utilizes the electrolyte itself as the functional material and all the device's constituent materials have demonstrated long-term stability in LIB electrolytes, thereby eliminating the conventional issue faced by flexible pressure sensors used for operando LIB sensing. To validate the long-term stability of the UIS device, a test is conducted by embedding it in the LIB with a heavy object placed on top, as depicted in Fig. 2j. By continuously monitoring and recording the outputs for more than 1 month, no significant changes are observed across both units. This demonstrates its potential for accurately monitoring the pressure of LIBs throughout their entire service life. In contrast, the outputs of conventional flexible pressure sensors encapsulated in Al-plastic film based on resistive, capacitive and iontronic mechanisms cannot maintain long-term stability due to the influence of penetrated solvents on the resistivity, dielectric constant or UAC of the functional materials, respectively, in a 30-day test, as depicted in Fig. 2k and Fig. S6. Specifically, the admittance output of the flexible resistive sensor using graphite/polymer composite as the functional material gradually attenuates over time, with only 1% remaining after 30 days, as illustrated in Fig. 2l. Furthermore, the gold electrode remains stable without any obvious crack or corrosion on the surface (Fig. S7).

### Instrument configuration for the operando pressure monitoring of LIBs

**Figure 3.**
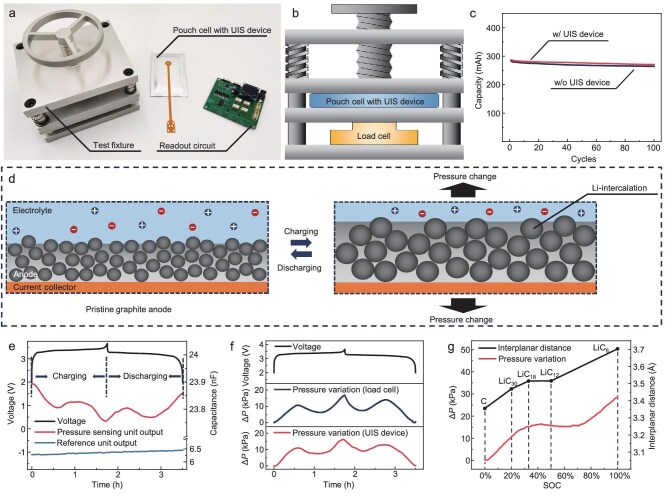
Operando pressure monitoring of LIBs using the UIS device. (a, b) Test set-up for the operando measurement of the pressure variations in the LIB using the UIS device. (c) Impact of UIS device on the aging performance of LIBs (tested at 0.5 C for 100 cycles). (d) Scheme of the mechanism of the electrode expansion during charging/discharging. (e) Capacitive outputs of the pressure sensing unit and the reference unit during a single charging/discharging process. (f) Comparison of the pressure variation detected using the UIS device (calculated from the data in Fig. 3e) with that detected by an external load cell, indicating a high degree of alignment on the pressure variation trend. (g) Interplanar distance of the graphite under different states of charging, explaining the volumetric change of the anode during charging.

The pressure variations of LIBs primarily stem from various electrode reactions that occur during charging/discharging processes (Fig. 2f). First, throughout the charging cycle, the graphite anode undergoes an expansion of ∼13% due to the intercalation of Li^+^ ions [38]. Additionally, as the LIB ages, the development and proliferation of the SEI layer on the anode surface leads to a notable increase in internal pressure. Most importantly, a high C-rate can potentially lead to the plating of Li dendrites on the surface instead of the intercalation of Li^+^ ions in the anode, substantially augmenting the anode's volume [39]. These electrode reactions result in distinct volume variations of the anode, which can be detected and differentiated by monitoring pressure changes. The test set-up for the operando measurement of the pressure variations in the LIB using the UIS device is illustrated in Fig. 3a. It comprises a test fixture, a UIS-device unified pouch cell and a readout circuit. The specialized test fixture has been designed to facilitate comparative testing between the UIS and an external load cell. The set-up involves clamping the pouch cell with the UIS and the load cell between two rigid plates, with a fixing device ensuring stable detection of the expansion force in the LIB, as shown in Fig. 3b and Fig. S8.

### Operando pressure monitoring of LIBs using the UIS device for state-of-charge (SOC) evaluation

At first, the influence of UIS devices on the performance of LIBs is evaluated. A comparative analysis is conducted to assess the capacity retention of LIBs with and without the UIS device over 100 charging/discharging cycles. As illustrated in Fig. 3c, it can be observed that both configurations exhibit nearly identical capacity retentions, thereby indicating that incorporating an UIS device does not have a detrimental effect on the battery's capacity or cycle life. This can be attributed to the following reasons: firstly, all components of the UIS device are derived from materials already utilized in LIBs. Specifically, the *in-situ* monitoring device leverages the ions of the battery electrolyte to form an electrical double layer at the device's electrodes, which does not introduce any potentially unstable substances, such as hydrogels or ionic gels commonly used in iontronic sensors, which could interfere with the electrochemical reactions of the LIB. Moreover, the UIS device monitors pressure changes caused by the volume variations of LIB electrodes, which represent the intrinsic changes occurring during battery operation. The extremely low thickness of the UIS device (∼100 μm) ensures that it does not exert excessive pressure on the LIB electrode, thereby avoiding any significant impact on the electrochemical reactions of the LIB.

As mentioned before, the UIS device is mainly used to record the pressure variation during the charging/discharging process for the analysis of electrode reactions. When the charging/discharging rates are relatively low, the expansion of the battery electrode primarily occurs due to Li^+^ intercalation into graphite, as illustrated in Fig. 3d [40]. Importantly, the expansion force is closely associated with the quantity of Li^+^ intercalated in the graphite anode, thus enabling its utilization for SOC evaluation. Figure 3e shows the operando capacitance variation curve monitored by the UIS device within a charging/discharging cycle. The data reveal that as the battery voltage increases from 2 V to 3.7 V during the charging process and then decreases back to 2 V during discharging, the capacitance of the pressure sensing unit undergoes a corresponding variation, while the reference unit exhibits subtle fluctuations in capacitance. Through the combination of the capacitive outputs of both units, the pressure variation curve of the battery throughout a charging/discharging cycle can be obtained, as depicted in Fig. 3f. As can be seen, there is an additional pressure peak during both the charging and discharging processes, which arises from the non-linear volumetric expansion characteristics of the graphite anode and the contraction of the cathode as Li^+^ intercalation/deintercalation [41]. The figure also shows a comparison between the pressure variation over time, measured by an external load cell, and that derived from the UIS device, demonstrating a high degree of alignment between them. This highlights that the UIS device is capable of accurately reflecting internal pressure changes for LIBs.

Figure 3g offers an explanation for the irregular pressure variation of the LIB during charging/discharging. The primary volumetric expansion is attributed to the increase in the interplanar lattice distance as Li^+^ ions intercalate into the graphite. At the onset of the charging process, graphite rapidly transitions to LiC_30_, causing a swift increase in the interplanar lattice distance and a corresponding rapid increase in pressure. As charging continues, LiC_30_ further transitions into LiC_18_ and LiC_12_, during which the interplanar lattice distance changes minimally. Concurrently, the sustained contraction of the cathode materials leads to a temporary decrease in internal pressure. However, toward the end of the charging process, the transition from LiC_12_ to LiC_6_ results in a significant increase in the interplanar lattice distance of graphite, culminating in a further rapid pressure increase which peaks at the end of the charging cycle. Given that the LIB operates as a reversible system, this process is mirrored during discharging [42].

### UIS device for Li dendrite detection during fast charging

**Figure 4.**
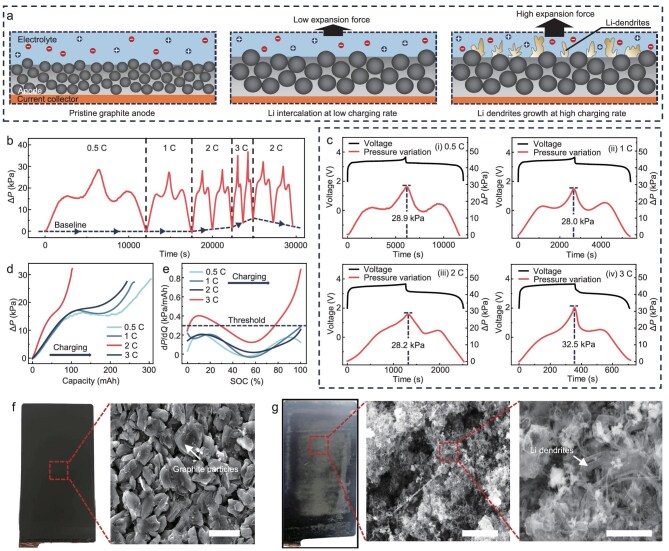
UIS device for Li dendrite detection during fast charging. (a) The principle of detecting Li dendrite growth based on pressure variation. The growth of Li dendrites will significantly increase the expansion force during charging. (b) Pressure variation of the LIB under continuous charging/discharging processes at charging rates of 0.5 C, 1 C, 2 C, 3 C and 2 C. (c) Detailed pressure variation detected by the UIS device in a single charging/discharging process at different charging rates. (d) The relationship between the real-time capacity and the pressure variation under different charging rates. (e) dP/dQ profiles under different charging rates. Specific regions within the dP/dQ profile during 3 C charging exceed the Li plating threshold, which can encompass all dP/dQ profiles at 0.5 C, 1 C and 2 C without Li dendrite growth. (f) Photograph and SEM images of the anode prior to 3 C charging (scale bar: 20 μm), and (g) illustrates the anode after 3C charging (scale bars: 20 μm and 5 μm). These images reveal the emergence of a compact layer of mossy deposits on the surface of the anode, accompanied by extensive Li dendrites in these regions as observed through SEM analysis.

Pressure measurement has been proven to be an effective and simple method for the early detection of Li dendrites [43,44]. In theory, when Li^+^ ions intercalate normally into the anode material, the volume change is minimal and significantly unleashes the battery capacity. Conversely, during fast charging, the formation of Li dendrites results in a substantial volume change, leading to a more significant increase in pressure [40], as shown in Fig. 4a. To demonstrate the efficacy of the UIS device for *in-situ* monitoring of Li dendrites in LIBs, the pressure variation curves of LIBs under different charging/discharging rates (0.5 C, 1 C, 2 C and 3 C) are examined, as depicted in Fig. 4b and c, and Fig. S9. Specifically, at 2 C and 3 C, there is a notable increase in peak pressure in the fully charged state and a clear elevation of valley pressure in the fully discharged state due to irreversible Li plating occurring on the anode (Fig. 4b). Moreover, at relatively low charging/discharging rates of 0.5 C and 1 C, the pressure variation curves exhibit a high degree of symmetry, indicating the reversible intercalation and deintercalation of Li^+^ ions. However, this symmetry is disrupted at the higher charging/discharging rates of 2 C and 3 C. Additionally, the pressure amplitudes in a single cycle increase due to the growth of extra Li dendrites at high charging rates, particularly at 3 C where a significantly higher pressure amplitude of 32.5 kPa is detected compared to around 28.5 kPa observed at low charging rates (Fig. 4c). The loss of symmetry in the pressure curves and the continuously increased valley pressure highlights the onset of irreversible Li plating, underscoring the sensitivity and capability of the UIS device in detecting these critical changes in real time.

A more quantitative approach to detect Li dendrites based on pressure involves analyzing the real-time change in cell pressure per unit of charge (dP/dQ) and comparing it with a threshold defined by the maximum dP/dQ during Li^+^ ion intercalation into the anode. Figure 4d and e depict the correlation between the internal pressure variation and battery capacity, as well as the relationship between dP/dQ and the SOC during a single charging process under different charging rates. The internal pressure significantly increases as the capacity of the LIB increases at a high charging rate of 3 C in comparison to 0.5 C, 1 C and 2 C. Additionally, specific regions within the dP/dQ profile during 3 C charging exceed the Li plating threshold, which can encompass all dP/dQ profiles at 0.5 C, 1 C and 2 C without Li dendrite growth. The optical and scanning electron microscopy (SEM) images of the anode before and after 3 C charging are compared to verify the growth of the Li dendrites, as depicted in Fig. 4f and g. Consequently, optical observation of Li dendrites aligns with analysis based on the detected pressure variation of the LIB, indicating that the UIS device can effectively detect the onset of Li dendrites in the LIB.

### UIS device for long-term LIB aging analysis

**Figure 5.**
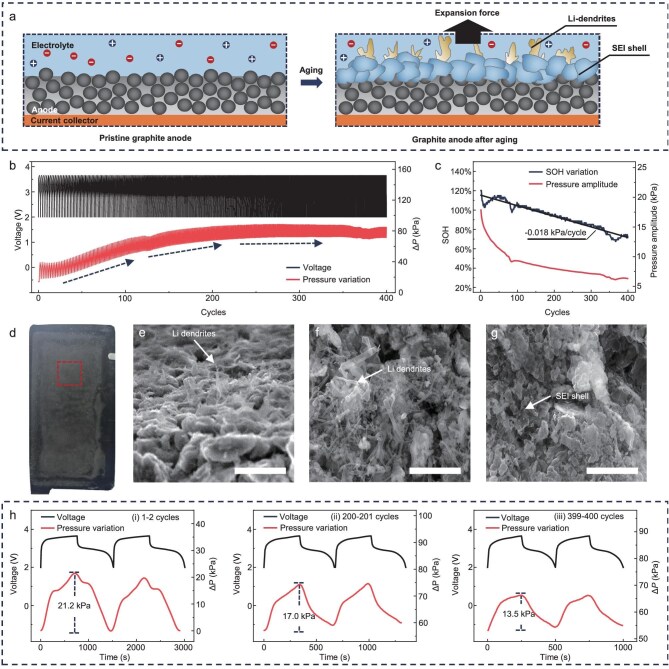
UIS device for long-term LIB aging analysis. (a) Mechanism scheme of the analysis of LIB aging based on pressure. The growth of the SEI shell and Li dendrites cause the variation of the expansion force in the LIB. (b) Voltage and pressure variation of the LIB during an accelerated aging experiment (3 C) involving 400 cycles. (c) Relationships between the number of cycles and the state of health (SOH) of the LIB and the pressure amplitude at each cycle. (d–g) Photographic and SEM images capturing the anode surface after 400 accelerated aging cycles. These images clearly demonstrate the presence of a substantial SEI layer on the anode surface along with the growth of Li dendrites, thus corroborating long-term pressure monitoring results obtained through the UIS device. Scale bar is 10 μm. All the SEM images are captured from the same electrode. (h) Pressure variations detected by the UIS device at different cycles, which can serve as characteristic parameters for assessing SOH, considering their distinct pressure amplitudes and waveforms.

With the aging of the LIB, the continual growth of irreversible Li dendrites and SEI shell will also influence the pressure variation during charging/discharging, as shown in Fig. 5a. To verify the capability of the UIS device for long-term stable monitoring of the LIB throughout its entire lifespan, an accelerated aging experiment involving charging/discharging for 400 cycles is conducted, with pressure variation curves monitored, as shown in Fig. 5b. Analysis of the pressure variation indicates a consistent increase in the average internal pressure of the LIB with an increasing number of cycles. This trend can be attributed to the irreversible expansion of the anode volume caused by the formation of additional SEI shell and Li dendrites during battery aging [45]. Consequently, there is an overall reduction in battery capacity because the growth of SEI shell and Li dendrites consumes the Li^+^ ions in the electrolyte, which can be assessed by examining its state of health (SOH), represented as the capacity retention (Fig. 5c) from 100% to 30% [46] (Fig. S10). This phenomenon is further evidenced by observing the decreasing peak-to-valley amplitude of pressure fluctuations in the single charging/discharge process, at a rate of −0.018 kPa/cycle, as the cycle number increases, due to continuous consumption of Li^+^ ions in electrolyte. The SEM images also prove the growth of Li dendrites and SEI shell after 400 cycles of aging, as shown in Fig. 5d–g.

It is worth noting that the UIS device consistently maintained excellent performance throughout the entire lifecycle of the LIB. From the pristine to the 400th cycle, the UIS device consistently provided precise and stable pressure measurements, as depicted in Fig. 5h. In summary, the UIS device exhibits remarkable potential for effectively monitoring battery aging characteristics and ensuring reliable performance over extended cycling life in LIBs. Moreover, the pressure mappings in the LIB after various charging/discharging cycles, as recorded by the UIS array, can illustrate the internal pressure distribution associated with the aging state of the battery. This information can provide more comprehensive results for SOH monitoring when integrated with deep learning algorithms, or facilitate theoretical research on electrochemical reactions within the system when combined with chemical analysis, as depicted in Fig. S11.

## CONCLUSION

By incorporating an *in-situ* UIS mechanism into the flexible pressure sensing system, we have successfully developed and implanted an encapsulation-free UIS device into LIBs for operando pressure monitoring. As a membrane device, the UIS device possesses all the advantages of conventional flexible pressure sensors. Moreover, since the battery electrolyte serves as the functional material of the device and all its components have demonstrated long-term stability within LIBs due to their shared building materials, the UIS sensor also exhibits exceptional stability in electrolytic environments. Consequently, it has maintained a stable output in pouch cells for over 800 hours without any signs of degradation. As a proof of concept, a pouch cell equipped with an UIS device is subjected to a fast-charging test ranging from 0.5 C to 3 C for the early detection of Li dendrites via pressure variations. Additionally, an accelerated aging test of 400 cycles is also conducted to analyze pressure variations during different cycles and assess the battery's health state. In summary, integrating an UIS device into operando physical-chemical event detection in LIBs could inspire self-sensing battery design in onboard applications that achieve functions such as battery health monitoring and safety pre-warning with compact architecture, low cost, high accuracy and long service life.

## MATERIALS AND METHODS

### Preparation of the UIS device

#### Preparation of the micro-structured PP film

The *in-situ* UIS device is characterized by an open structure exposed to the environmental electrolyte and utilizes micro-structured PP film as the pressure-sensitive material. The PP material, originally serving as the building material of the separator in LIBs, exhibits exceptional long-term stability within the corrosive LIB electrolyte. Typically, commercially purchased biaxially oriented PP film (50 μm thickness, purchased from Guangzhou Bosheng packaging company) has a smooth surface; however, for creating the pressure-sensitive interface, a surface roughening process is employed. The micro-structure on the PP film is created through a hot-pressing process by pressing the film on a coarse sandblasting metal plate (with a Ra of 28 μm) at 150°C and 10 MPa for 40 minutes (Fig. S12). Subsequently, it is laser cut to match the size of the electrode and expose the region corresponding to the reference unit in the middle (1.7 mm in diameter) (Fig. S13). It is important to note that the elastic modulus of the PP film, surface roughness of the sandblasting plate, and hot-pressing parameters, as well as the diameter of the exposed region, will impact the sensing properties of this device (Fig. S14). The parameters listed here have been optimized through testing according to the sensitivity, sensing range, repeatability and stability of the UIS device.

#### Fabrication of flexible electrodes

The fabrication of flexible electrodes for the UIS device involves creating a patterned conductive electrode using traditional etching techniques. The layers, from bottom to top, consist of a 25-um-thick PI substrate layer, a 12-um-thick copper circuit layer, a 2-um-thick nickel-plating layer, a 600-nm-thick gold-plating layer and a 25-um-thick PI protective layer. The gold metal is directly exposed in the electrolyte and protects the copper and nickel underneath from electrochemical corrosion. It is important to note that the top PI protective layer only exists in edge regions of the device (Fig. S15). The preparation of the flexible electrodes follows the mature technology of flexible printed circuits (FPCs) and is processed by Shenzhen Liansheng Electronic Industrial Co., LTD.

#### Assembly of the UIS device

The flexible electrode is aligned with the micro-structured PP layer, and then they are temporarily bonded together at the substrate edge using a mold under hot pressure. It is important to avoid applying pressure on the electrode region. It should be ensured that only the micro-structured PP layer comes into contact with the pressure sensing unit, while the reference unit remains exposed to air.

#### Assembly of the LIB with the UIS device

The LIB pouch cell is utilized in this study to verify the function of the UIS device, as the force in the LIB can effectively transmit through its flexible encapsulation, allowing for a reliable comparison between the UIS output and externally measured pressure. Specifically, the fabricated pouch cell follows the conventional battery architecture wherein cathode-coated current collectors, separators, and anode-coated current collectors are laminated within an aluminum-plastic (Al-plastic) film encapsulation with injected organic electrolyte. Here, a widely used commercial LIB, employing lithium iron phosphate (LiFePO₄) as the cathode, graphite as the anode, and a mixed solution of lithium hexafluorophosphate (LiPF₆) in ethylene carbonate (EC)/methyl ethyl carbonate (MEC) as the electrolyte, is chosen, aiming to demonstrate the universality of the UIS device. The pouch cell is assembled with a graphite anode (coated on a copper current collector) and a LiFePO_4_ cathode (coated on an aluminum current collector), both punched into uniform sizes of 4.1 cm × 8.0 cm and 4 cm × 7.5 cm, respectively. The negative/positive (N/P) ratio of the active materials is controlled within the range of 1.03–1.15, while the separator and organic electrolyte consist of glass fiber and LiPF_6_ dissolved in a mixed organic solvent of MEC/EC. Comprising three pieces of LiFePO_4_ cathodes, four pieces of graphite anodes, and six separators, the anodes and cathodes are stacked in a laminated manner before being encapsulated with Al-plastic film. The tabs for the anode and cathode are made from Ni foils and Al foils respectively. The UIS device is securely positioned between the outermost anode and the package, while the electrode connector extends beyond the packaging (Figs S16 and S17). Following a 3-day drying process in a vacuum oven, ∼10 mL of electrolyte is injected into the pouch before sealing. It should be noted that lug adhesives are employed to ensure a strong adherence between the Al-plastic film and the electrode of the UIS device.

### Operando pressure monitoring of LIBs using the UIS device

To validate the feasibility of operando pressure monitoring using the UIS device, a test set-up comprising a test fixture and a readout circuit has been developed. The test fixture has been designed to facilitate comparative testing between the UIS device and an external load cell. The fixture involves clamping the pouch cell with the UIS device between two rigid plates, ensuring uniform preload around 50 kPa by applying a specific force using four springs at each corner of the top plates. Simultaneously, the bottom plate is directly placed on a load cell to enable transmission of expansion force from the pouch cell (Fig. S8). The readout circuit is utilized to switch the channel of the pressure sensing unit and the reference unit to the Inductance, Capacitance, Resistance (LCR) meter, enabling alternate measurement of their capacitive outputs (Fig. S18). The simultaneous measurement will result in signal crosstalk due to the shared electrolyte as functional material for both units. A battery testing system (Lanhe CT 3004A) is employed for charging or discharging at various rates, and an LCR meter is used to record the UIS device's outputs concurrently. Besides the LiFePO_4_-based LIB, another widely used type of LIB, namely Nickel-Cobalt-Manganese (NCM), was also employed to demonstrate the feasibility of the UIS device for operando pressure monitoring in LIBs, as shown in Fig. S19.

## Supplementary Material

nwaf151_Supplemental_File

## References

[1] Cano ZP, Banham D, Ye S et al. Batteries and fuel cells for emerging electric vehicle markets. Nat Energy 2018; 3: 279–89. 10.1038/s41560-018-0108-1

[2] Tu S, Zhang B, Zhang Y et al. Fast-charging capability of graphite-based lithium-ion batteries enabled by Li_3_P-based crystalline solid–electrolyte interphase. Nat Energy 2023; 8: 1365–74. 10.1038/s41560-023-01387-5

[3] Zhao L, Wang T, Zuo F et al. A fast-charging/discharging and long-term stable artificial electrode enabled by space charge storage mechanism. Nat Commun 2024; 15: 3778. 10.1038/s41467-024-48215-2 38710689 PMC11074309

[4] Huang CJ, Thirumalraj B, Tao HC et al. Decoupling the origins of irreversible coulombic efficiency in anode-free lithium metal batteries. Nat Commun 2021; 12: 1452. 10.1038/s41467-021-21683-6 33664259 PMC7933276

[5] Cheng XB, Zhang R, Zhao CZ et al. Toward safe lithium metal anode in rechargeable batteries: a review. Chem Rev 2017; 117: 10403–73. 10.1021/acs.chemrev.7b00115 28753298

[6] Colclasure AM, Tanim TR, Jansen AN et al. Electrode scale and electrolyte transport effects on extreme fast charging of lithium-ion cells. Electrochim Acta 2020; 337: 135854. 10.1016/j.electacta.2020.135854

[7] Huang J, Boles ST, Tarascon JM. Sensing as the key to battery lifetime and sustainability. Nat Sustain 2022; 5: 194–204. 10.1038/s41893-022-00859-y

[8] Blanquer LA, Marchini F, Seitz JR et al. Optical sensors for operando stress monitoring in lithium-based batteries containing solid-state or liquid electrolytes. Nat Commun 2022; 13: 1153. 10.1038/s41467-022-28792-w 35241673 PMC8894478

[9] Li R, Li W, Singh A et al. Effect of external pressure and internal stress on battery performance and lifespan. Energy Storage Mater 2022; 52: 395–429. 10.1016/j.ensm.2022.07.034

[10] Müller V, Scurtu RG, Richter K et al. Effects of mechanical compression on the aging and the expansion behavior of Si/C-composite|NMC811 in different lithium-ion battery cell formats. J Electrochem Soc 2019; 166: A3796–805. 10.1149/2.1121915jes

[11] Zhu S, Yang L, Wen J et al. In operando measuring circumferential internal strain of 18650 Li-ion batteries by thin film strain gauge sensors. J Power Sources 2021; 516: 230669. 10.1016/j.jpowsour.2021.230669

[12] Gervillié-Mouravieff C, Boussard-Plédel C, Huang J et al. Unlocking cell chemistry evolution with operando fibre optic infrared spectroscopy in commercial Na(Li)-ion batteries. Nat Energy 2022; 7: 1157–69. 10.1038/s41560-022-01141-3

[13] Mei W, Liu Z, Wang C et al. Operando monitoring of thermal runaway in commercial lithium-ion cells via advanced lab-on-fiber technologies Nat Commun 2023; 14: 5251. 10.1038/s41467-023-40995-3 37640698 PMC10462619

[14] Luo Y, Abidian MR, Ahn JH et al. Technology roadmap for flexible sensors. ACS Nano 2023; 17: 5211–95. 10.1021/acsnano.2c12606 36892156 PMC11223676

[15] Rogers JA, Someya T, Huang Y. Materials and mechanics for stretchable electronics. Science 2010; 327: 1603–7. 10.1126/science.1182383 20339064

[16] Hirvikorpi T, Vähä-Nissi M, Mustonen T et al. Atomic layer deposited aluminum oxide barrier coatings for packaging materials Thin Solid Films 2010; 518: 2654–8. 10.1016/j.tsf.2009.08.025

[17] Lange J, Wyser Y. Recent innovations in barrier technologies for plastic packaging—a review. Packag Technol Sci 2003; 16: 149–58. 10.1002/pts.621

[18] Chang Y, Wang L, Li R et al. First decade of interfacial iontronic sensing: from droplet sensors to artificial skins. Adv Mater 2021; 33: 2003464. 10.1002/adma.202003464 33346388

[19] Tang J, Zhao C, Luo Q et al. Ultrahigh-transparency and pressure-sensitive iontronic device for tactile intelligence. npj Flex Electron 2022; 6: 54. 10.1038/s41528-022-00162-y

[20] Nie B, Xing S, Brandt JD et al. Droplet-based interfacial capacitive sensing. Lab Chip 2012; 12: 1110–18. 10.1039/c2lc21168h 22311169

[21] Cheng Y, Guo C, Li S et al. Aquatic skin enabled by multi-modality iontronic sensing. Adv Funct Mater 2022; 32: 2205947. 10.1002/adfm.202205947

[22] Li S, Pan N, Zhu Z et al. All-in-one iontronic sensing paper. Adv Funct Mater 2019; 29: 1807343. 10.1002/adfm.201807343

[23] Li B, Ge R, Du W et al. iWood: an intelligent iontronic device for human-wood interactions. Adv Funct Mater 2024; 34: 2314190. 10.1002/adfm.202314190

[24] Sun B, Zhang Z, Xu J et al. Composite separator based on PI film for advanced lithium metal batteries. J Mater Sci Technol 2022; 102: 264–71. 10.1016/j.jmst.2021.06.040

[25] Wang F, Ke X, Shen K et al. A critical review on materials and fabrications of thermally stable separators for lithium-ion batteries. Adv Mater Technol 2022; 7: 2100772. 10.1002/admt.202100772

[26] Corti CW, Holliday RJ. Commercial aspects of gold applications: from materials science to chemical science. Gold Bull 2004; 37: 20–6. 10.1007/BF03215513

[27] Heikenfeld J, Jajack A, Rogers J et al. Wearable sensors: modalities, challenges, and prospects. Lab Chip 2018; 18: 217–48. 10.1039/C7LC00914C 29182185 PMC5771841

[28] Lux SF, Lucas IT, Pollak E et al. The mechanism of HF formation in LiPF_6_ based organic carbonate electrolytes. Electrochem Commun 2012; 14: 47–50. 10.1016/j.elecom.2011.10.026

[29] Costa V, Nohales A, Félix P et al. Enhanced polyurethanes based on different polycarbonatediols. J Elastomers & Plastics 2013; 45: 217–38.

[30] Mencer HJ, Gomzi Z. Swelling kinetics of polymer-solvent systems. Eur Polym J 1994; 30: 33–6. 10.1016/0014-3057(94)90229-1

[31] Maksimov AV, Molina M, Maksimova OG et al. Prediction of swelling of polypropylene separators and its effect on the lithium-ion battery performance. ACS Appl Polym Mater 2023; 5: 2026–31. 10.1021/acsapm.2c02074

[32] Kang D, Pikhitsa PV, Choi YW et al. Ultrasensitive mechanical crack-based sensor inspired by the spider sensory system. Nature 2014; 516: 222–6. 10.1038/nature14002 25503234

[33] Chen Z, Ming T, Goulamaly MM et al. Enhancing the sensitivity of percolative graphene films for flexible and transparent pressure sensor arrays. Adv Funct Mater 2016; 26: 5061–7. 10.1002/adfm.201503674

[34] Xia F, Xu SA. Effect of surface pre-treatment on the hydrophilicity and adhesive properties of multilayered laminate used for lithium battery packaging. Appl Surf Sci 2013; 268: 337–42. 10.1016/j.apsusc.2012.12.091

[35] Park BK, Jeong YK, Yang SY et al. Deterioration behavior of aluminum pouch film used as packaging materials for pouch-type lithium-ion batteries. J Power Sources 2021; 506: 230222. 10.1016/j.jpowsour.2021.230222

[36] Chatham H . Oxygen diffusion barrier properties of transparent oxide coatings on polymeric substrates. Surf Coat Technol 1996; 78: 1–9. 10.1016/0257-8972(95)02420-4

[37] Guo Z, Fan Y. Heat seal properties of polymer–aluminum–polymer composite films for application in pouch lithium-ion battery. RSC Adv 2016; 6: 8971–9. 10.1039/C5RA27097A

[38] Louli AJ, Ellis LD, Dahn JR. Operando pressure measurements reveal solid electrolyte interphase growth to rank Li-ion cell performance. Joule 2019; 3: 745–61. 10.1016/j.joule.2018.12.009

[39] Wang C, Ma Z, Wang Y et al. Failure prediction of high-capacity electrode materials in lithium-ion batteries. J Electrochem Soc 2016; 163: A1157. 10.1149/2.0251607jes

[40] Huang W, Ye Y, Chen H et al. Onboard early detection and mitigation of lithium plating in fast-charging batteries. Nat Commun 2022; 13: 7091. 10.1038/s41467-022-33486-4 36402759 PMC9675798

[41] Missyul A, Bolshakov I, Shpanchenko R. XRD study of phase transformations in lithiated graphite anodes by Rietveld method. Powder Diffr 2017; 32: S56–62. 10.1017/S0885715617000458

[42] Nitta N, Wu F, Lee JT et al. Li-ion battery materials: present and future. Mater Today 2015; 18: 252–64. 10.1016/j.mattod.2014.10.040

[43] Che Y, Hu X, Lin X et al. Health prognostics for lithium-ion batteries: mechanisms, methods, and prospects. Energy & Environm Sci 2023; 16: 338–71.

[44] Lu X, Tarascon JM, Huang J. Perspective on commercializing smart sensing for batteries. eTransportation 2022; 14: 100207. 10.1016/j.etran.2022.100207

[45] Xiong R, Pan Y, Shen W et al. Lithium-ion battery aging mechanisms and diagnosis method for automotive applications: recent advances and perspectives. Renew Sustain Energy Rev 2020; 131: 110048. 10.1016/j.rser.2020.110048

[46] Zeng J, Liu S. Research on aging mechanism and state of health prediction in lithium batteries. J Energy Storage 2023; 72: 108274. 10.1016/j.est.2023.108274

