# Supplementary material for: Unified iontronic sensing for operando monitoring of physical-chemical events in lithium-ion batteries
Source: Natl Sci Rev. 2025 Apr 25;12(6):nwaf151. doi: 10.1093/nsr/nwaf151 (PMC12121480; doi:10.1093/nsr/nwaf151)
Supplement: nwaf151_Supplemental_File [file nwaf151_supplemental_file.pdf]

Supplementary Data

Unified iontronic sensing for operando monitoring of physical-chemical events in lithium-ion batteries

Yu Chang*[#], Yu Cheng[#], Rui Jia[#], Ruojiang Wang[#], Qing Xu, Lanqing Gong, Yike Wei, Bin Tang, Chenhui Guo, Bin Sun, Xingmin He, Xueyan Li, Lili Gong, Hong Ye, Xiaoyang Wang, Yitao Dai, Mingdong Dong, Yongbing Tang*, Fan Zhang*, Peng Tan*and Tingrui Pan*

Corresponding authors: yuchang@ustc.edu.cn, tangyb@siat.ac.cn, fan.zhang1@siat.ac.cn, pengtan@ustc.edu.cn, tingrui@ustc.edu.cn

The PDF file includes:

Supplementary Text 1 to 2
Supplementary Figures 1 to 22
Supplementary Table 1

Supplementary Text 1

Mathematical derivations for mechanical response of the pressure sensing unit

For an iontronic pressure sensor based on a super-capacitive interface, the capacitive output C of the sensor can be described as:

$$C = C_0 \times A \tag{1}$$

Where C_0 represents the unit area capacitance of the super-capacitive interface, which is a constant for given material species, ion concentration, driving signal and environmental condition. A is the contact area of the super-capacitive interface [1-3]. It`s worth noting that the real electric double-layer is complicated and Eq. 1 is a simplified description that works well for calculating the capacitive output of the super-capacitive interface.

The capacitive output C_P of the pressure sensing unit is determined by the capacitance of the electrode/electrolyte interface, thus it can be defined as:

$$C_P = C_0 \times (A_P - A_C) \tag{2}$$

Where A_P represents the total surface area of the electrode, while A_C denotes the effective true contact area between the electrode and the micro-structured PP film. Since C_0 is a constant that can be measured through the reference unit, A_P remains constant as it was prepared. Therefore, in order to theoretically predict capacitive output accurately, it is crucial to calculate A_C.

Since all the solid surfaces are rough on the microscale, the contact between objects thus occurs at discrete spots produced by the mechanical contact of asperities on the two surfaces [4-6]. Therefore, the true contact area of two solid materials is a small fraction of the nominal contact area. Contact load will cause the advance on the true contact area via increasing the contacted asperities number and causing the compression deformation of the contacted asperities [4, 5]. As a result, the contact between the electrode and the micro-structured PP film under pressure for the UIS device can be categorized into the contact of the asperities at the interface, as shown in **Figure S20** [7].

The theoretical relationship between the true contact area in a rough surface, of which the size of its surface asperities is randomly distributed, and the load in the contact of nominally flat surfaces (smooth surfaces) has been developed according to Hertz contact theory[4]. The true contact area A_C and the load F applied to the interface can be expressed as:

$$A_C = \pi(\eta\beta\sigma)A_P \cdot e^{-\frac{d}{\sigma}} \tag{3}$$

$$F = \pi^{\frac{1}{2}}(\eta\beta\sigma)E`(\frac{\sigma}{\beta})^{\frac{1}{2}}A_P \cdot e^{-\frac{d}{\sigma}} \tag{4}$$

$$\frac{1}{E`} = \frac{1-v_1^2}{E_1} + \frac{1-v_2^2}{E_2} \tag{5}$$

η is the density of the asperities on the surface, β is the radius of the asperity summit, σ is the standard deviation of the height distribution of the asperities, d is the distance of the reference plane in the rough surface with the smooth surface, E_1, E_2, are Young`s modulus of the materials along the interface, while v_1 and v_2 are their Passion's rates respectively. Importantly, the derivation of Eq. 3 and Eq. 4 follows the assumption that the heights of the asperities follow an exponential distribution, which is a fair approximation when the deformation is small (lower than uppermost 25% of the asperities of the surface has been deformed, which happens under relative low pressure or the Young`s modulus of the material is large). A larger deformation will cause the deviation to the theoretical prediction. As a result, the relationship between the true contact area and the Load is:

$$\mathrm{A}_C = \frac{\pi^{\frac{1}{2}}(\frac{\beta}{\sigma})^{\frac{1}{2}}F}{E^{`}} \tag{6}$$

Considering the relationship between the pressure P and the load applied:

$$\mathrm{P} = \frac{F}{A_P} \tag{7}$$

The true contact area at different pressure can be expressed as:

$$\mathrm{A}_C = \mathrm{A}_P \cdot \frac{\pi^{\frac{1}{2}}(\frac{\beta}{\sigma})^{\frac{1}{2}}P}{E^{`}} \tag{8}$$

Eq. 8 can be simplified into:

$$\mathrm{A}_C = \mathrm{k}^{`}\mathrm{A}_P \cdot \frac{P}{E^{`}} \tag{9}$$

Whereas k` represents the influences of the surface geometrical parameters of the micro-structured PP film and can be treated as a constant for given surface roughening parameters. By introducing Eq. 9 to Eq. 2, the capacitance C_P of the pressure sensing unit under external pressure applied can be described as:

$$C_P = C_0 \times (A_P - \mathrm{k}^{`}\mathrm{A}_P \cdot \frac{P}{E^{`}}) \tag{10}$$

Considering k` and A_P are all constant, Eq. 10 can also be simplified as:

$$C_P = C_0 \times (A_P - \mathrm{k}\frac{P}{E^{`}}) \tag{11}$$

Here k represents k`×A_P.

It is crucial to emphasize that the theoretical equation primarily aims to determine the factors influencing the output of the pressure sensing unit for performance optimization. According to Eq. 11, the capacitive output is predominantly determined by the UAC of the electrolyte and the modulus of the micro-structured PP film. Other parameters, such as the entire surface area of the electrode and k, remain constant once device preparation is completed. Considering the intricacy of super-capacitive interfaces, approximated surface asperity distribution and challenge to calculate parameter k, there may be significant deviations between theoretical equations and experimental results. However, the theoretical equation still provides qualitative insights into analyzing device outputs under different conditions.

Supplementary Text 2

Additional Materials and Methods Section

Characterizations of the UIS device

Mechanical properties characterization of the polymers

The polymer films are immersed in the LIB electrolyte contained within a glass bottle and sealed using parafilm. The stress-strain curves of the polymers are determined by conducting tests using a motorized force tester (ESM 303, Mark-10) in accordance with the standard ASTM D3039.

Measurement setup of the UIS device

The capacitance-to-contact pressure curves of pressure sensing unit of the UIS device is tested by recording the capacitance readouts of the unit immersed in the electrolyte under increased contact pressure applied by the motorized force tester in a rate of 0.5mm/min. The capacitive outputs are measured using an Inductance-Capacitance-Resistance (LCR) digital bridge (TH2829C, Tonghui Inc.) with scanning frequency and voltage of 50 kHz and 500 mV respectively (**Fig. S21**). The electrolyte used here is an aquatic LIB electrolyte of 3% Bis(trifluoromethane)sulfonimide lithium salt (LiTFSI) in water, for the commonly used LIB organic electrolyte is highly instable in the atmosphere. The response/reset time and the repeatability of the pressure sensing unit are measured via applying periodical pressure of 1 Hz through an electromagnetic relay. A signal generator (AFG1022, Tektronix) is used to apply a periodical square wave of ±5 V to the electromagnetic relay. The readouts of the sensor are amplified through operating amplifier circuitry and recorded by a data acquisition card with an extremely high sampling rate of 1 M/s (DAQ, NI USB-6361, NI instruments) or recorded by LCR respectively.

Long-term stability of the UIS device and the encapsulated flexible sensor in LIBs

The long-term stability of the UIS device is tested by encapsulating it in a pouch cell and subjecting it to a weight of 500g. Subsequently, the battery is maintained at a constant temperature of 25˚C for over one month, during which the LCR continuously records the capacitive outputs of both the pressure sensing unit and the reference unit at a rate of 1 point per minute. The encapsulated flexible sensor is prepared based on the piezoresistive mechanism. The flexible sensor consists of an interdigital electrode, which is fabricated using the same process as that of the UIS device, and it is in contact with a pressure-sensitive film prepared by printing carbon paste (EMS Cl-2022) on 50 μm PET film. The performance of the flexible sensor heavily relies on the mechanical and electrical properties of the carbon paste, which comprises conductive graphite powder and polyester binder. Subsequently, the flexible sensor is encapsulated in a 113 μm thick Al-plastic film (Suzhou YDS new materials Co., LTD), followed by sealing it within a LIB with a weight of 500g placed on the top (**Fig. S22**). The resistive output of the sensor is recorded using a multimeter (TH1942, Tonghui Inc.).

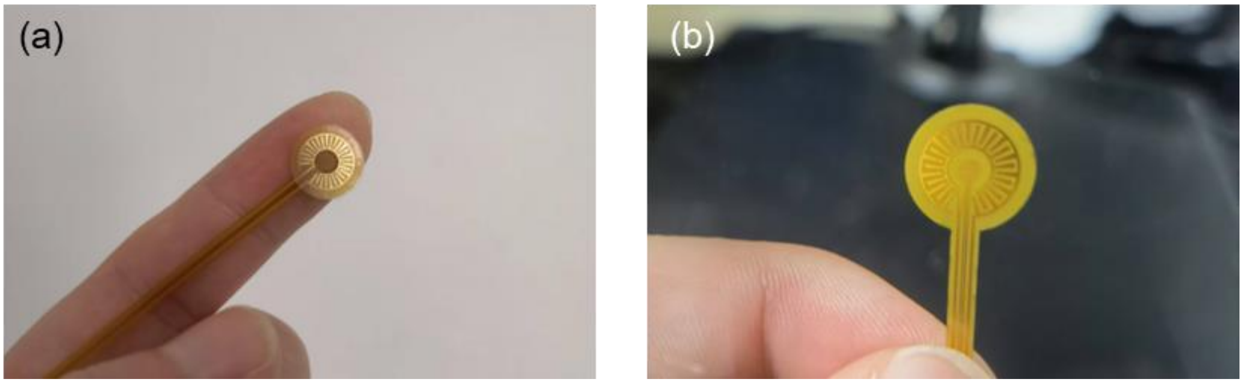

Figure S1. The photographs of the UIS device, which utilizes a commercial FPC with a thickness of 50 μm (Au/Nickel/Copper metal pattern on a 25 μm flexible polyimide substrate) as the electrode, illustrate the size, thickness, and flexibility of the device.

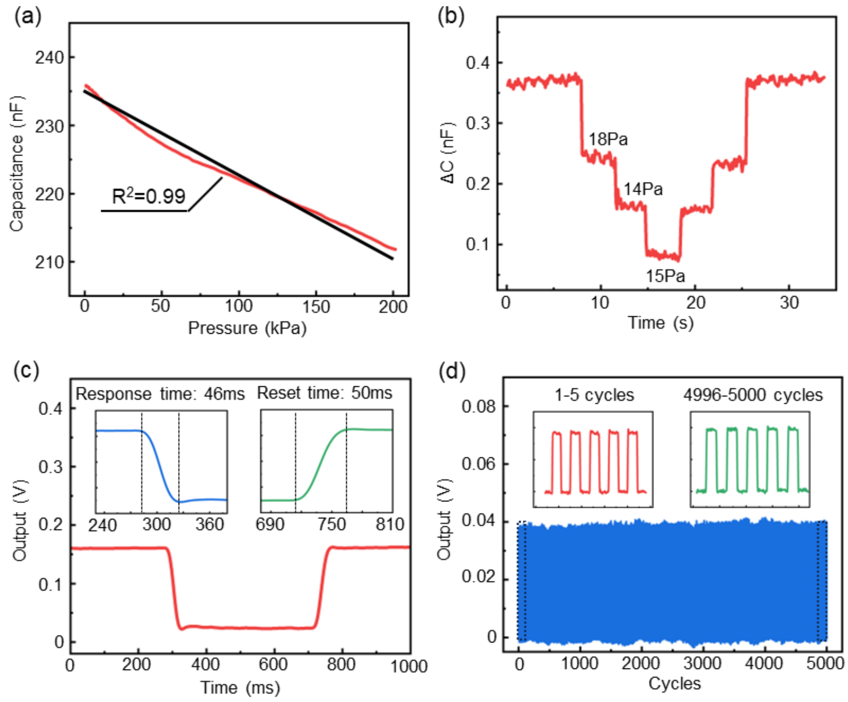

Figure S2. a) The capacitance-to-pressure curve of the pressure sensing unit below 200kPa. **b)** The pressure resolution of the pressure sensing unit. **c)** The response time of the pressure sensing unit. **d)** The repeatability of the pressure sensing unit.

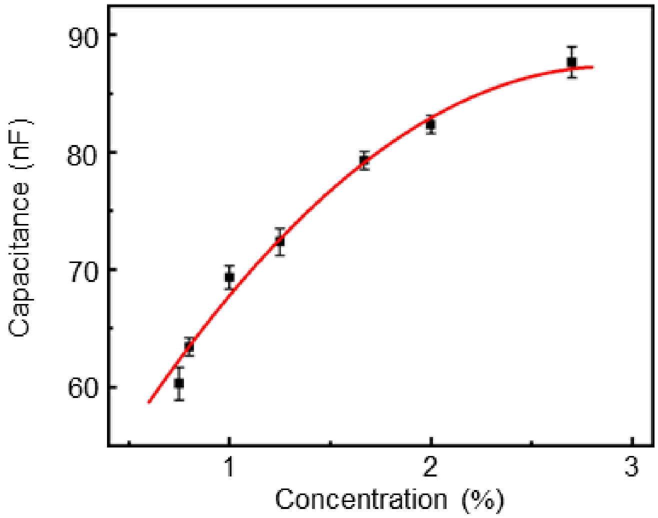

Figure S3. The relationship between the concentration of the electrolyte and the capacitive output of the reference unit, data are mean ± SD, *N*=3.

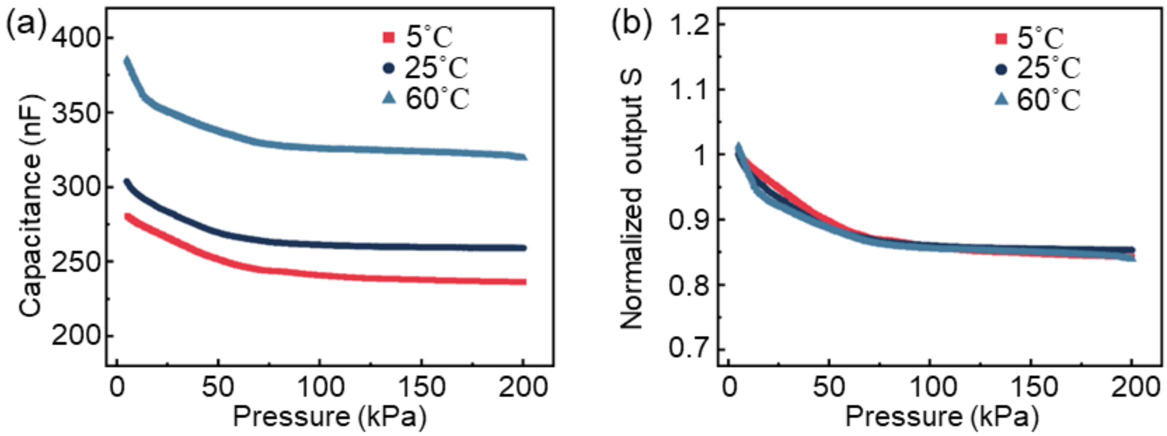

Figure S4. a) The pressure-to-capacitance curves of the pressure sensing unit of the UIS device in the electrolyte at various temperatures ranging from 5 ℃ to 60 ℃. **b)** The compensation for the variation in ionic conductivity of the electrolyte at different temperatures is achieved by combining the outputs of the pressure sensing unit (C_P) and the reference unit (C_R), demonstrating that the normalized output of the device exhibits minimal sensitivity to environmental temperature.

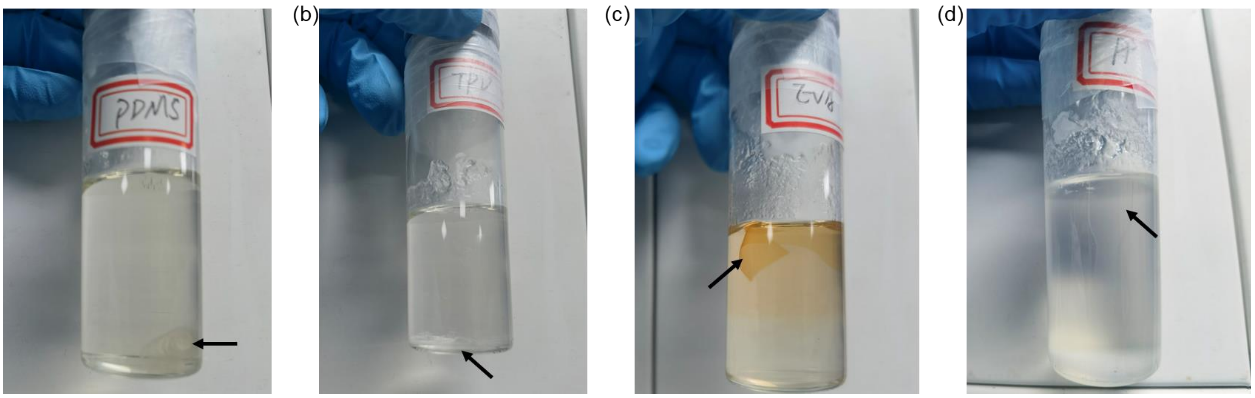

Figure S5. The samples of **a)** PDMS, **b)** TPU, **c)** EVA, and **d)** PP after immersion in LIB organic electrolyte for one month. Except PP, the other samples underwent certain levels of morphological alterations or chemical reactions.

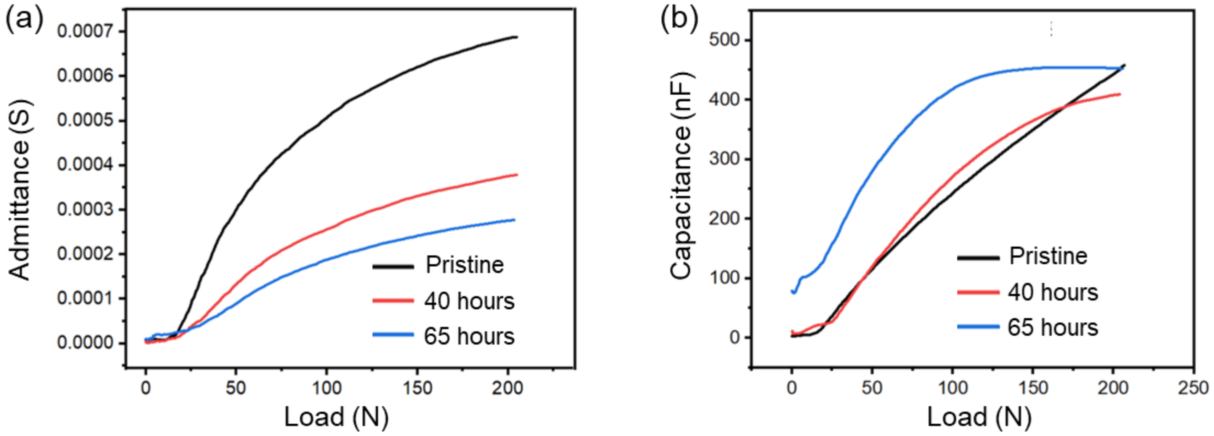

Figure S6. a) The mechanical response of the flexible pressure sensor, which utilizes carbon paste as the pressure sensitive film, was tested after immersion in the organic electrolyte of LIBs for various durations. The sensitivity of the pressure sensor gradually decreases over time due to the infiltration of organic solvents through the Al-plastic film. **b)** The flexible pressure sensor utilizes an ionic gel film as the pressure sensitive layer, while all other components remain unchanged. The composition of the ionic gel film includes 1g of polyvinylidene fluoride (PVDF), 0.2g of LiTFSI, 0.8g of a mixed solvent consisting of MEC/EC in a weight ratio of 1:1, and 0.6g of silicon dioxide powder (1000 mesh). This film is prepared by screen printing a paste dissolved in 4g acetone onto a PET film. It is worth noting that the binder (PVDF) used in this particular type of ionic gel is the same with the anode material and employs the same solvent as the electrolyte to minimize the potential impact on its properties caused by solvent penetration. As can be seen, the mechanical response of the sensor remains stable for up to 40 hours of immersion; however, it gradually changes thereafter. This test aims to demonstrate that the commonly used flexible pressure sensor encapsulated in Al-plastic film fails to maintain long-term stability in LIB, regardless of its piezoresistive or iontronic mechanism.

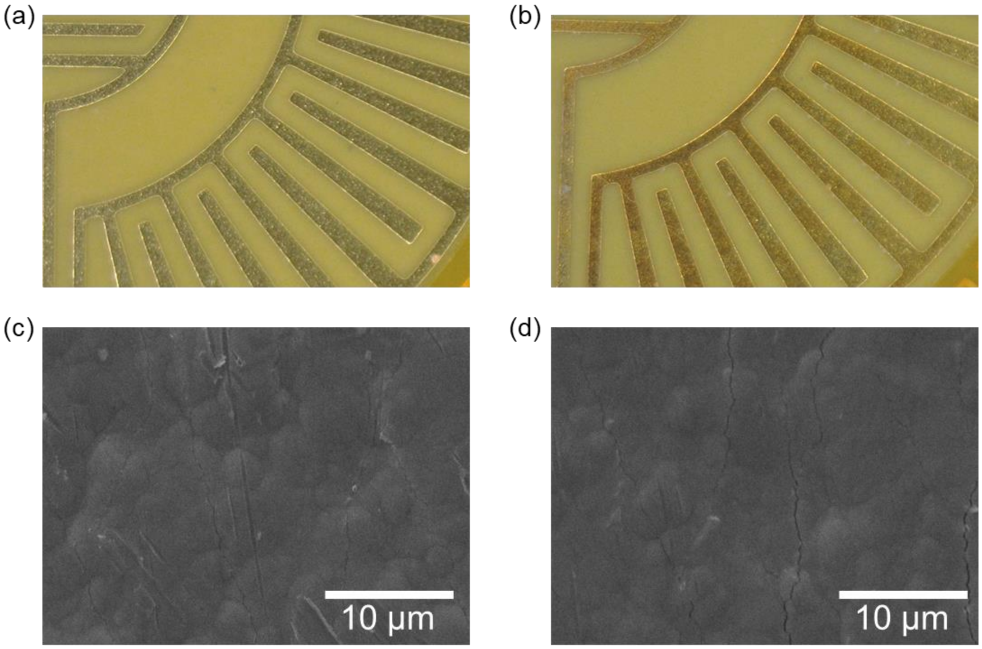

Figure S7. The electrode's appearance **a)** before and **b)** after one month of immersion in the LIB organic electrolyte, along with its measurement by an LCR. A subtle alteration in the gold circuit's color can be observed, but no obvious changes on capacitive output happens. The SEM images **c)** before and **d)** after immersion exhibit no discernible changes at the micro-scale level.

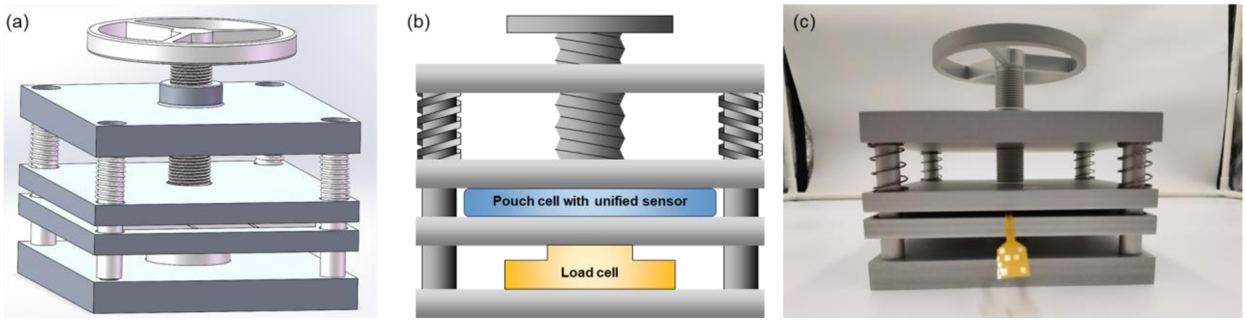

Figure S8. The design of the test fixture. The specialized fixture has been designed to facilitate comparative testing between the UIS device and an external load cell. The setup involves clamping the pouch cell with UIS and the load cell between two rigid plates, with a fixing device ensuring stable detection of the expansion force in the LIB.

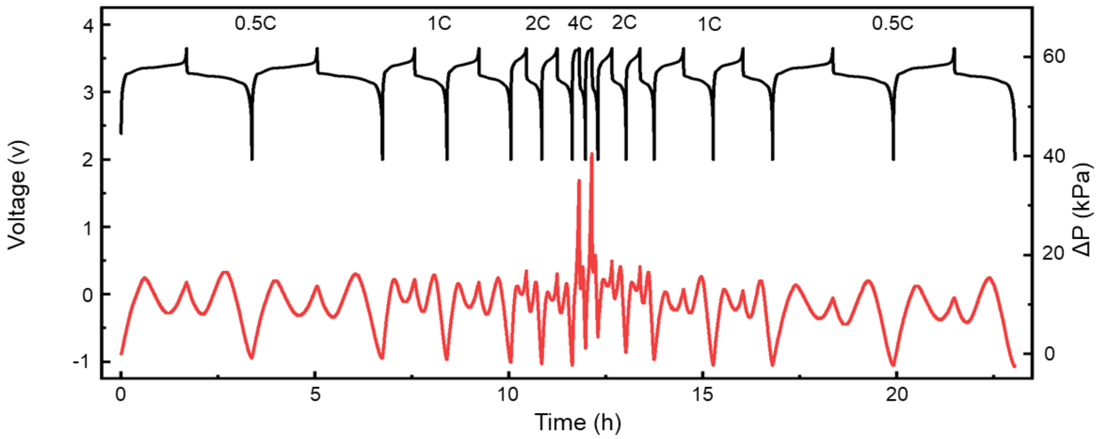

Figure S9. More results about the pressure variations of the LIB in different charging rates. As a comparison, the results are tested from the load cell. Still, charging at high rates leads to a more significant increase on the pressure baseline and the pressure amplitude.

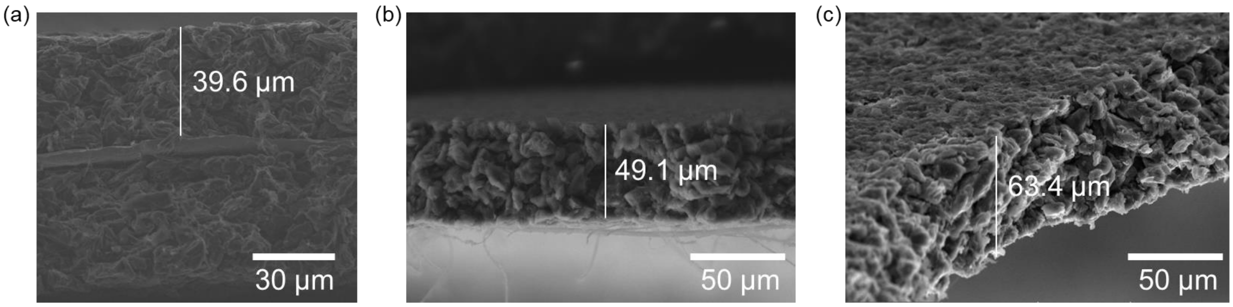

Figure S10. The cross-sectional views of the graphite layer on the anode **a)** in its pristine state, **b)** when fully charged, and **c)** after aging for 400 cycles are presented. Evidently, the thickness increases as lithium ion intercalates into the graphite, and a further thickness increase happens after aging due to deposit growth during repeated charging/discharging.

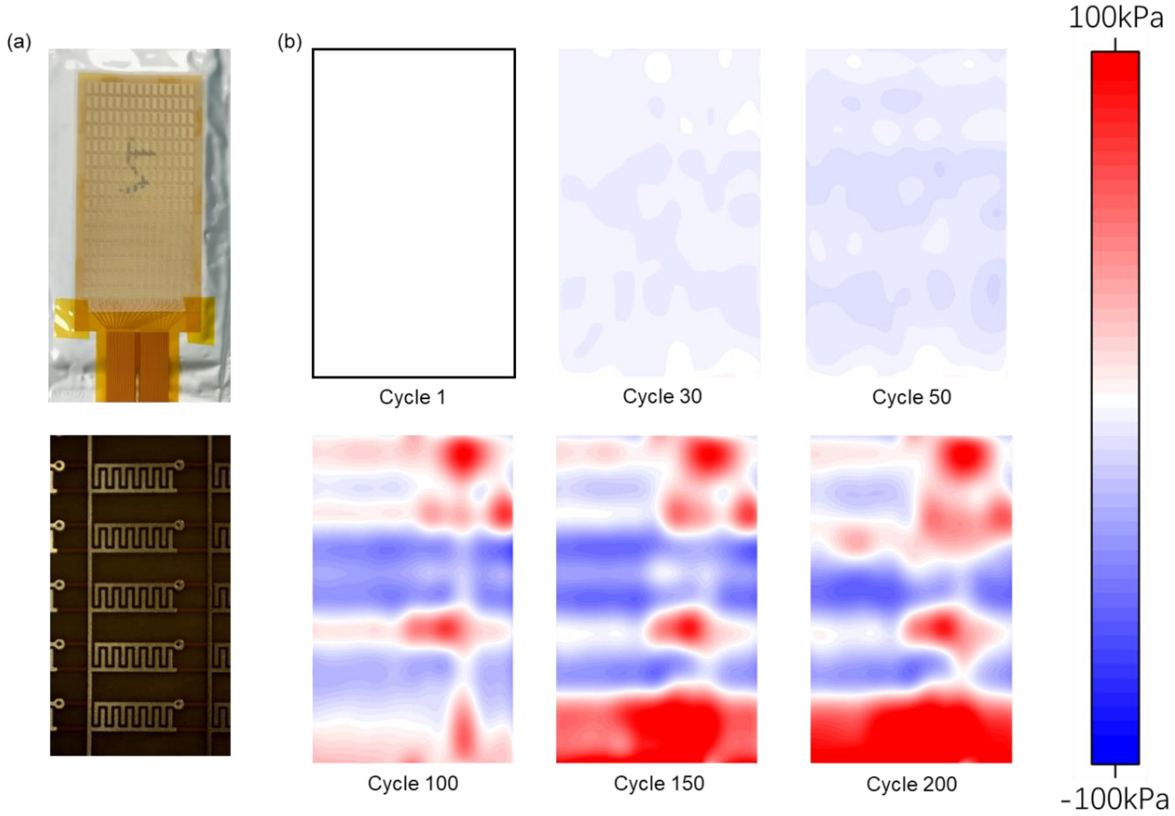

Figure S11. a) The design of the UIS device capable of detecting pressure distribution. **b)** Pressure distributions recorded by the UIS device for the LIB after various charging/discharging cycles.

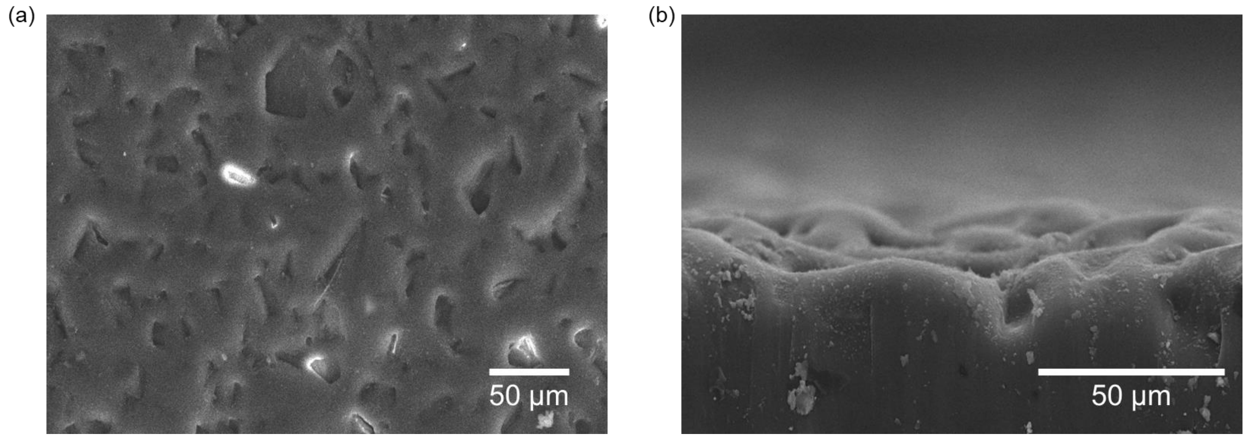

Figure S12. The SEM images of the micro-structured PP film, demonstrating a randomly distributed asperities on the surface.

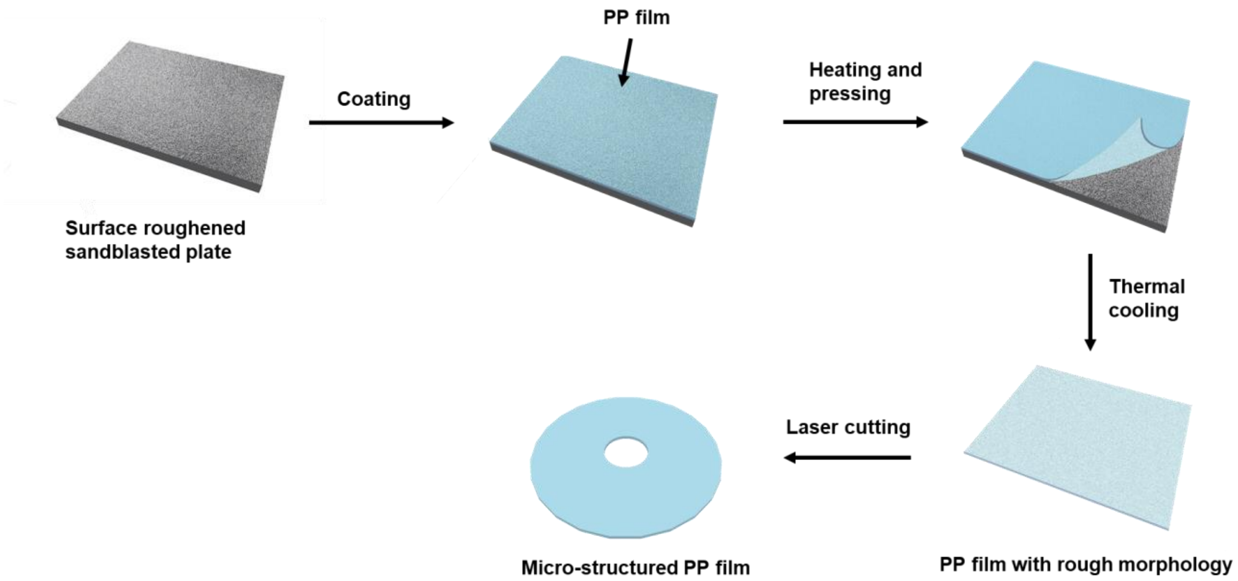

Figure S13. The preparation process of the micro-structured PP film.

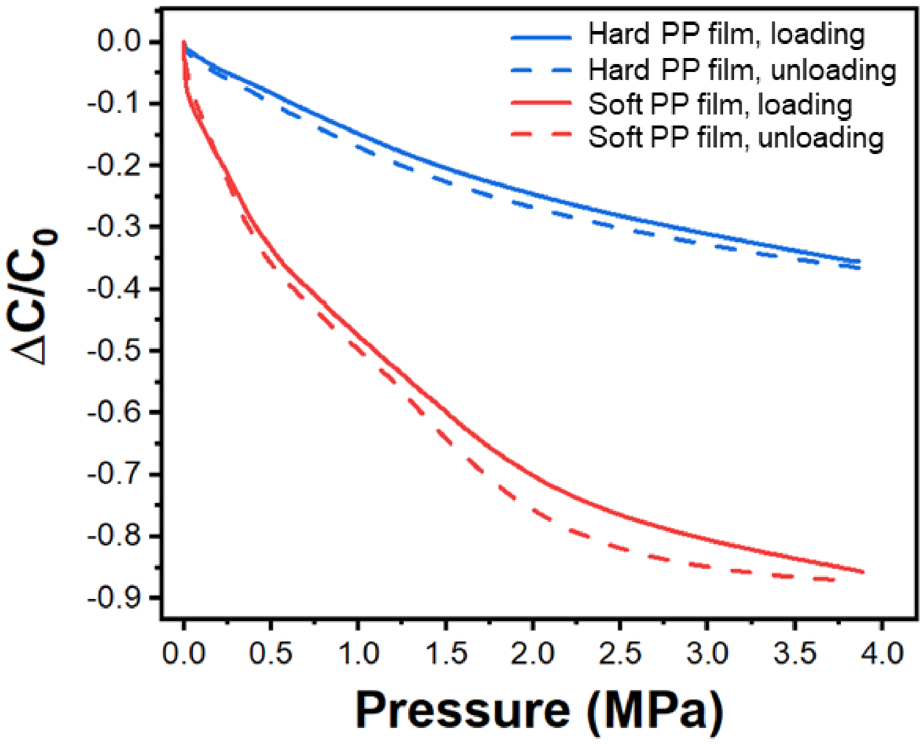

Figure S14. The capacitance-to-pressure curves of the pressure sensing units using micro-structured PP films with different Young's moduli. It is observed that the pressure sensing unit employing a softer PP film (with a Young's modulus of approximately 150 MPa) demonstrates higher sensitivity compared to the one utilizing a harder PP film (with a Young's modulus of around 600 MPa), fitting well with the theoretical prediction. However, the former one demonstrates greater hysteresis (15%) than that of the latter one (5%). Considering both the comprehensive sensing performance and long-term stability, the harder PP film is chosen for the preparation of the UIS device.

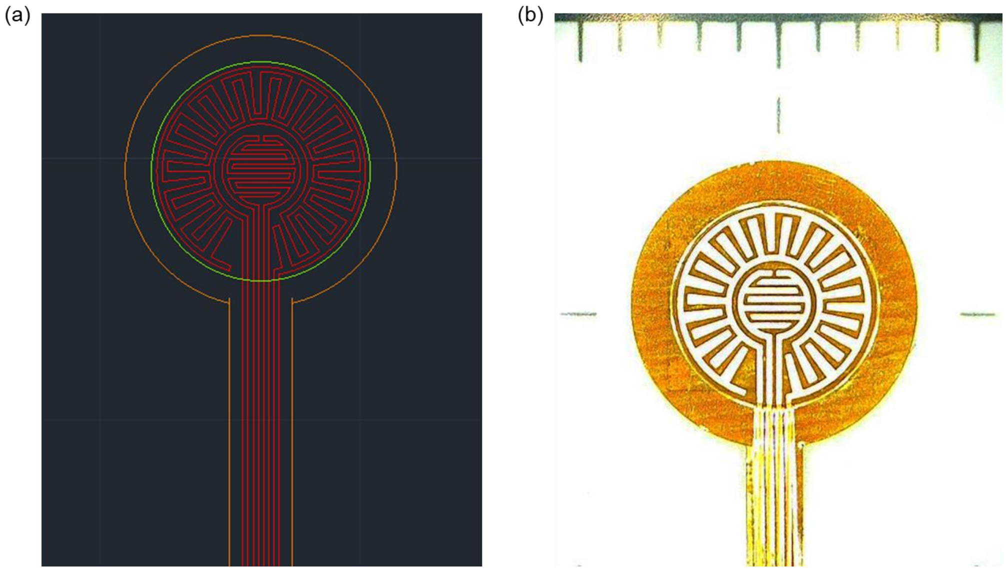

Figure S15. a) The circuit design of the electrode for the UIS device. **b)** The photo of the electrode, demonstrating two independent interdigital electrodes for pressure sensing unit and the reference unit.

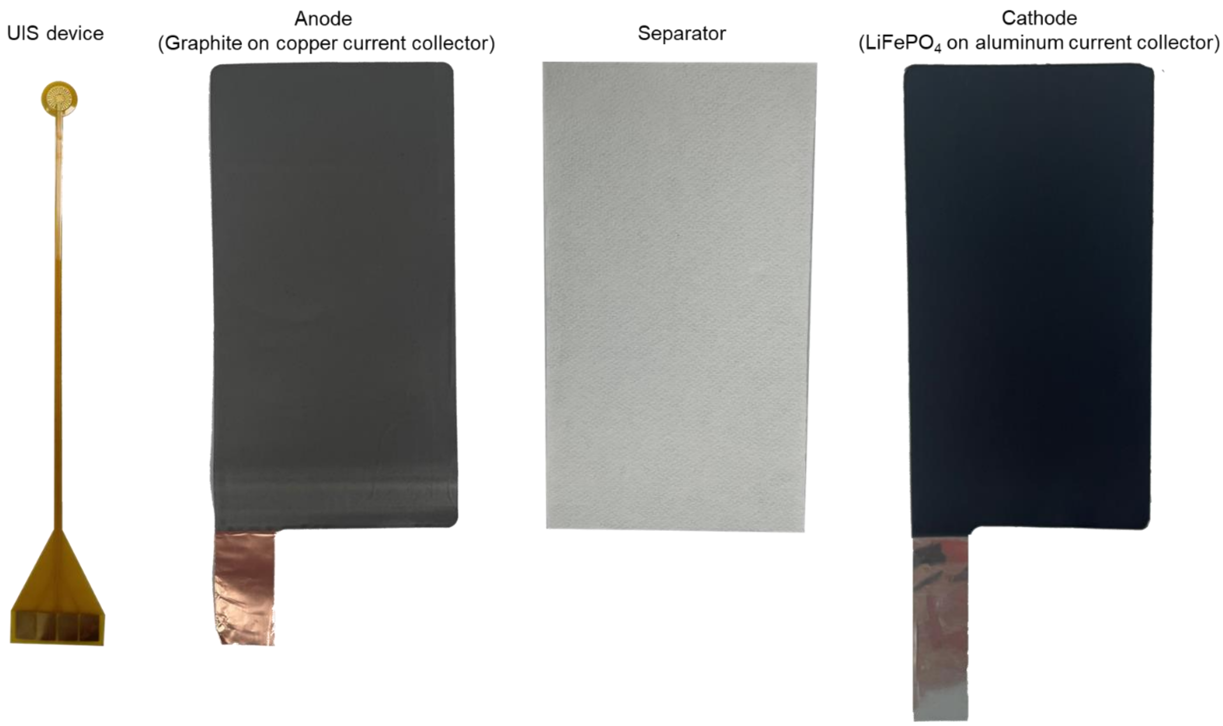

Figure S16. The photos of the components building the intelligent unified battery.

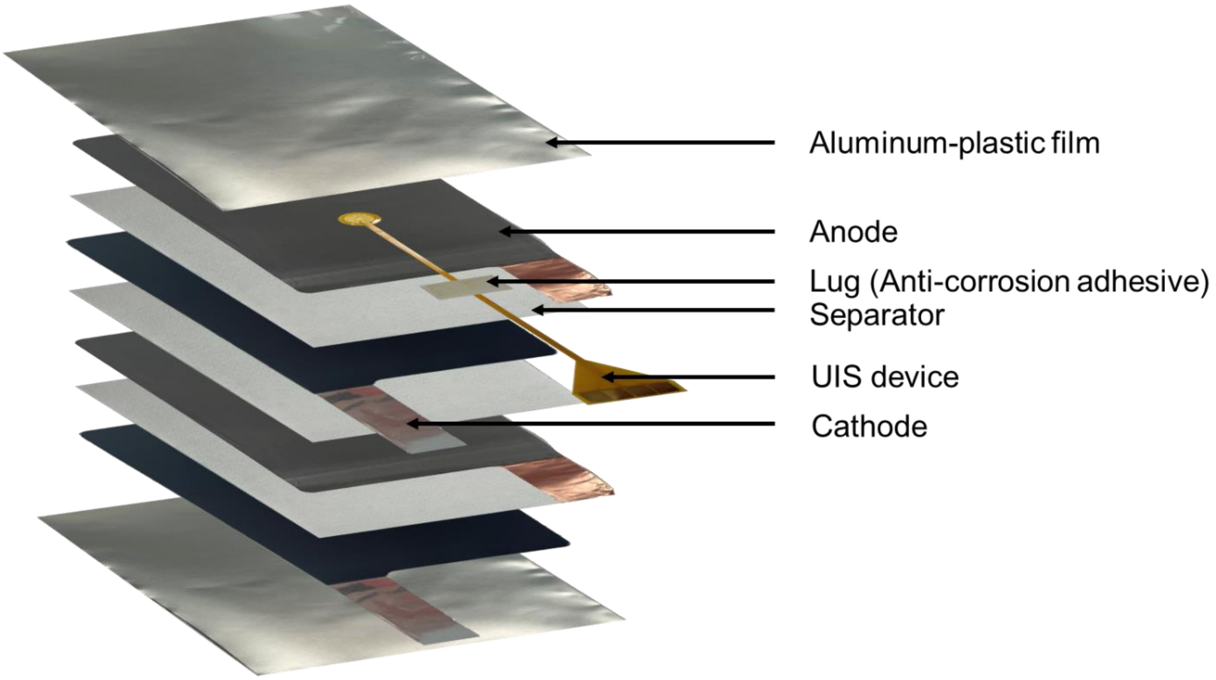

Figure S17. The exploded view of the intelligent unified battery.

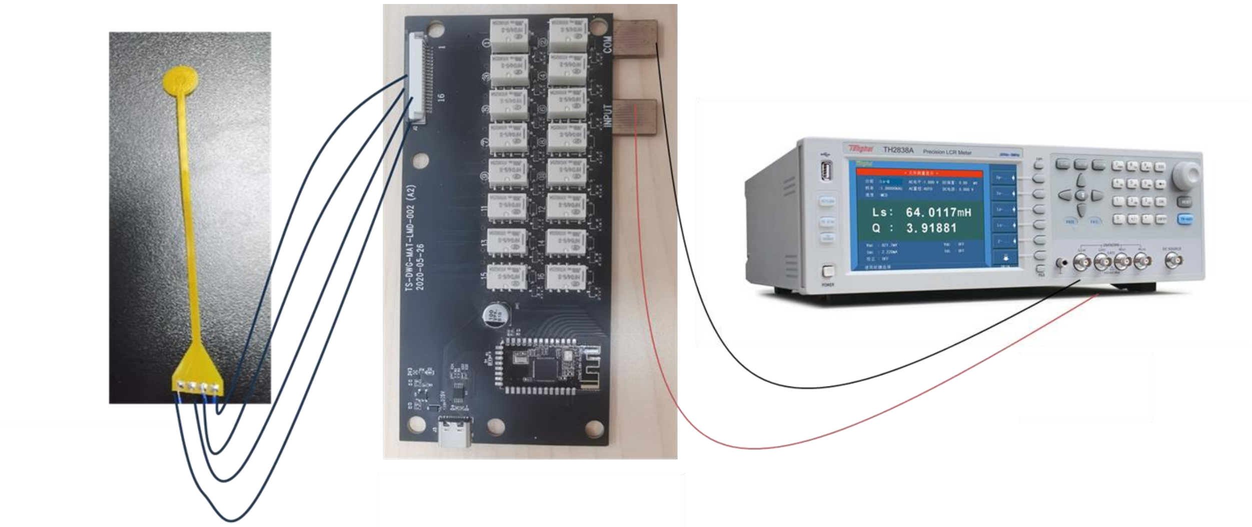

Figure S18. The readout circuitry for switching the channel (pressure sensing unit or reference unit) linked to the LCR meter, enabling alternate measurement of their capacitive outputs. Because the simultaneous measurement will result in signal crosstalk due to the shared electrolyte as functional material for both units.

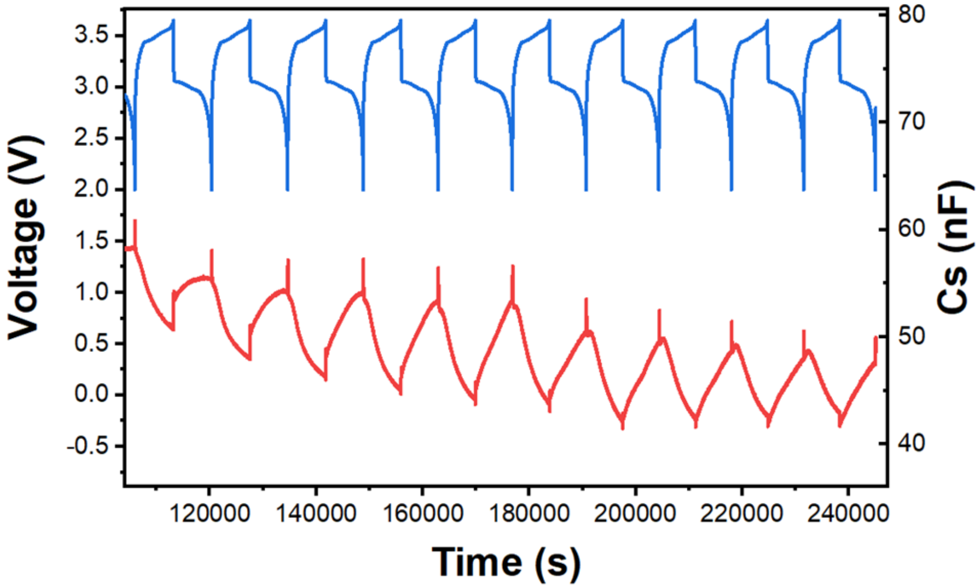

Figure S19. The operando pressure measurement using UIS device in a Nickel-Cobalt-Manganese (NCM) LIB, which is another type of widely used commercial LIB.

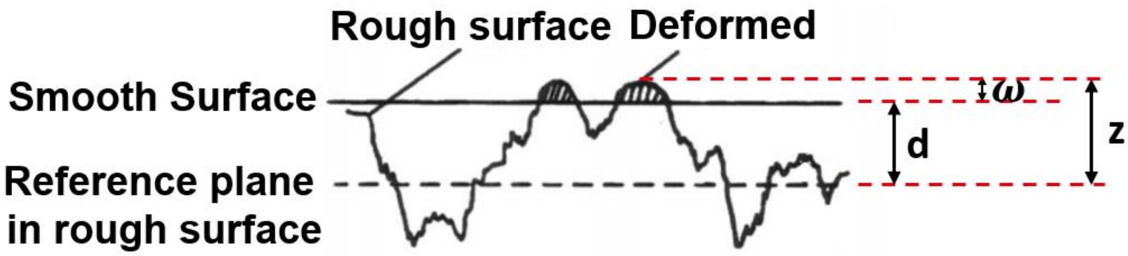

Figure S20. The simplified model of the contact between a rough surface with a nominally flat surface. d refers to the distance of the reference plane in rough surface with the smooth surface, while z means the height of the micro asperity and ω is the depth of the micro asperity entering the smooth surface [4].

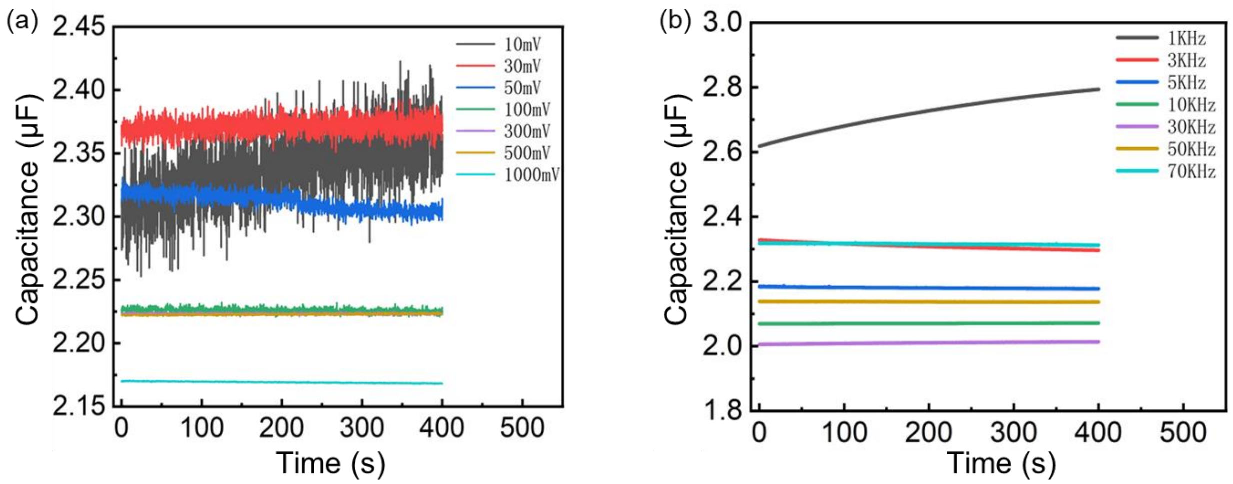

Figure S21. Capacitive outputs of the reference unit under different driving frequency and voltage. These tests aim to optimize the driving frequency and voltage for a higher sensitivity of the device, meanwhile the fluctuation and shift should be as low as possible for decreasing the noise and instability. Through comprehensive comparisons, the driving frequency of 50kHz and voltage of 500mV with lowest fluctuation and shift are selected.

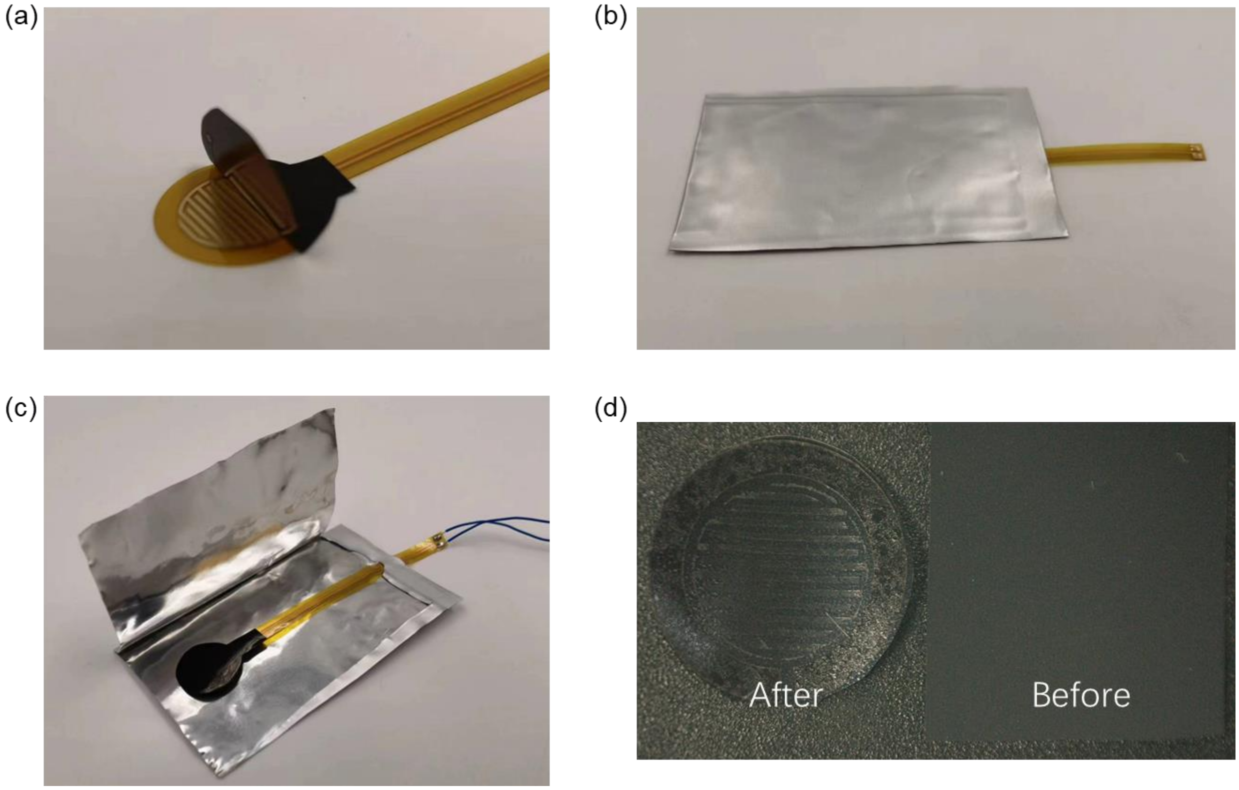

Figure S22. The encapsulated flexible pressure sensor for the comparison with the UIS device on long-term stability. **a)** The photo of the flexible pressure sensor, which consists of an interdigital electrode and a pressure-sensitive film made from carbon paste. **b), c)** The photo of the flexible pressure sensor encapsulated in an Al-plastic film, followed by sealing the entire package in a LIB. **d)** The pressure-sensitive film before and after being sealed in a LIB for one month, which reveals obvious traces of electrode-induced plastic deformation under pressure. Additionally, changes in color and morphology are observed on these traces of carbon paste, possibly resulting from organic solvent adsorption and electrochemical reactions with the electrode.

Table S1. Permeabilities of commonly used flexible materials in packaging[8].

Film	Oxygen permeability at 23˚C 50% or 0% RH $cm^3/(m^2 \cdot day \cdot atm)$	Water vapor permeability at 23˚C 85% RH $g/(m^2 \cdot day)$
Poly (ethylene terephthalate) (PET, 1mm)	1-5	0.5-2
Polypropylene (PP, 1mm)	50-100	0.2-0.4
Polyethylene (PE, 1mm)	50-200	0.5-2
Polystyrene (PS, 1mm)	100-150	1-4
Poly (vinyl chloride) (PVC, 1mm)	2-8	1-2
Poly (ethylene naphtholate) (PEN, 1mm)	0.5	0.7
Polyamide (PA, 1mm)	0.1-1(dry)	0.5-10
Poly (vinyl alcohol) (PVA, 1mm)	0.02(dry)	30
Ethylene vinyl alcohol (EVOH, 1mm)	0.001-0.01(dry)	1-3
Poly (vinylidene chloride) (PVDC, 1mm)	0.01-0.3	0.1
Aluminum-plastic film (PET 12 μm /Aluminum 9 μm/PE 50 μm)	~0*	~0**
Aluminum coated film (PET 12 μm /Vacuum-deposited aluminum layer /PE 50 μm)	1-2	0.1-0.5

*Almost zero, the product standard is lower than 10^{-3}~10^{-1} $cm^3/(m^2 \cdot day \cdot atm)$

** Almost zero, the product standard is lower than 10^{-6}~10^{-4} $g/(m^2 \cdot day)$

References

1. Y. Chang, L. Wang, R. Li, Z. Zhang, Q. Wang, J. Yang, C. F. Guo, T. Pan, First Decade of Interfacial Iontronic Sensing: From Droplet Sensors to Artificial Skins. *Advanced Materials* 2021, **33**, 2003464.
2. J. Tang, C. Zhao, Q. Luo, Y. Chang, Z. Yang, T. Pan, Ultrahigh-transparency and pressure-sensitive iontronic device for tactile intelligence. *npj Flex Electron* 2022, **6**, 54.
3. B. Nie, S. Xing, J. D. Brandt, T. Pan, Droplet-based interfacial capacitive sensing. *Lab Chip* 2012, **12**, 1110.
4. Greenwood, James A., and JB Pl Williamson. "Contact of nominally flat surfaces." Proceedings of the royal society of London. Series A. Mathematical and physical sciences 1966, **1442**, 300.
5. Timsit S. Electrical contact resistance: properties of stationary interfaces//Electrical Contacts-1998. Proceedings of the Forty-Fourth IEEE Holm Conference on Electrical Contacts (Cat. No. 98CB36238). IEEE, 1998: 1-19.
6. Li L, Song W, Zhang G, et al. An electrical contact resistance model including roughness effect for a rough MEMS switch. *Journal of Micromechanics and Microengineering* 2012, **22**, 115023.
7. Huang Y, Chen Y, Fan X, et al. Wood derived composites for high sensitivity and wide linear - range pressure sensing. *Small* 2018, **14**, 1801520.
8. J. Lange, Y. Wyser, Recent innovations in barrier technologies for plastic packaging—a review. *Packag Technol Sci* 2003, **16**, 149–158.
